# Paired single-cell profiling reveals epithelial–stromal remodeling after ablative fractional carbon dioxide laser treatment in keloids

**DOI:** 10.3389/fimmu.2026.1992388

**Published:** 2026-10-28

**Authors:** Dongxian Lin, Li Duan, Bincheng Wang, Xueying Chen, Yuchen Cao, Yuanyuan Xu, Shanbaga Zhao, Lianzhao Wang

**Affiliations:** 1 Department of Cicatrix Minimally Invasive Treatment Center, Plastic Surgery Hospital, Chinese Academy of Medical Sciences and Peking Union Medical College, Beijing, China; 2 Department of Surgical Biotechnology, UCL Division of Surgery and Interventional Science, University College London, London, United Kingdom

**Keywords:** ablative fractional carbon dioxide laser, fibroblast, keloid, keratinocyte, single-cell RNA sequencing, wound healing

## Abstract

**Background:**

Keloids are fibroproliferative scars, but the cellular responses to ablative fractional carbon dioxide laser (AFCO2) treatment remain poorly defined. This study aimed to characterize treatment-associated epithelial, stromal and immune remodeling in patient-matched human keloid tissue.

**Methods:**

We jointly reprocessed four previously reported untreated hypercellular-zone PRE specimens and four newly generated patient-matched POST specimens obtained 35 days after AFCO2 treatment from the same patients. All specimens were processed using the same Singleron Matrix/GEXSCOPE single-cell platform. Patient-aware pseudobulk differential expression, continuous transcriptional-program analysis, gene-set enrichment, trajectory and regulatory modeling, and cell–cell communication inference were integrated with independent reanalysis of GSE320017 and quantitative multiplex immunofluorescence and CD74 immunohistochemistry. The patient was the biological and inferential unit.

**Results:**

After quality control, 74,937 cells were retained. A KRT6-dominant keratinocyte program increased in all four patients; KRT6B increased by 4.44 log2 units (false-discovery rate, 2.65 × 10^−5^), with concordant increases in KRT6A and KRT16. Fibroblasts shifted toward an interferon/inflammatory program and away from a homeostatic extracellular-matrix program, whereas myeloid/APC cells showed lower HLA-DRA, C3 and MHC-II-associated antigen-presentation programs. Nine patient-supported mural-to-dendritic-cell or mural-autocrine communication axes weakened after treatment. Quantitative tissue imaging showed that CK6-family-positive area within CK14-defined epithelium increased from 2.70% to 65.84%, MMP10-positive area overlapping CK14-defined epithelium increased from 8.49% to 25.92%, and CD74-positive cell density decreased from 2,530.35 to 695.50 cells/mm². The independent hypertrophic-scar dataset showed context-dependent rather than uniform AFCO2 responses.

**Conclusions:**

AFCO2 treatment was associated with coordinated epithelial, stromal and immune remodeling in human keloids, centered on a KRT6-dominant epithelial program, fibroblast-state redistribution and attenuation of an MHC-II-associated tissue context. These patient-consistent findings are exploratory and do not establish causal mechanisms.

## Introduction

Keloids are fibroproliferative scars that extend beyond the original wound boundary and can cause pain, pruritus, functional restriction and substantial psychosocial burden. Their persistence reflects dysregulated wound repair rather than a simple excess of collagen: chronic inflammation, altered mechanotransduction, epithelial stress, fibroblast heterogeneity and abnormal extracellular-matrix (ECM) turnover interact across spatially distinct tissue compartments (1–7, 17). Although current management combines surgery, corticosteroids, pressure, silicone, radiotherapy and energy-based procedures, recurrence and variable response remain common (1–3, 17).

Ablative fractional carbon dioxide laser (AFCO2) treatment can remodel scar tissue through controlled thermal ablation and subsequent re-epithelialization (18–22). Clinical and histological studies support improvement in texture, pliability and thickness in selected hypertrophic and traumatic scars, but the cellular programs associated with AFCO2 treatment remain incompletely resolved (22–25). This uncertainty is particularly relevant to keloids, which differ from hypertrophic scars in growth pattern, inflammatory persistence and dermal organization. Most mechanistic studies rely on cross-sectional comparisons, cultured fibroblasts or animal wounds, whereas patient-matched human tissue sampled before and after treatment is uncommon.

Single-cell transcriptomics has revealed that fibroblasts comprise multiple context-dependent states with distinct inflammatory, matrix-producing and spatial functions (5–16). Keratinocytes also actively shape repair: wound-edge cells induce KRT6/KRT16/KRT17 programs, alter adhesion and migration, and communicate with stromal and immune compartments (27, 28). Matrix metalloproteinases, including MMP10, can remodel provisional matrix and influence keratinocyte organization, but their role must be interpreted in the context of tightly regulated repair rather than simple matrix destruction (29, 30). Together, these observations argue that treatment responses should be examined as coordinated epithelial–stromal-immune remodeling rather than as a fibroblast-only process.

We previously profiled infiltrating and hypercellular zones from four untreated anterior-chest keloids and identified marked regional immune-stromal heterogeneity (31). In the present study, the previously reported hypercellular-zone specimens served as PRE baselines; the infiltrating-zone specimens were not included. We jointly reprocessed these baseline samples with four newly generated POST specimens obtained from the same patients using the same sampling and single-cell platform. Patient-aware pseudobulk models, continuous transcriptional-program analysis, trajectory and regulatory modeling, and intercellular communication inference were used to define treatment-associated remodeling. We then independently reprocessed a hypertrophic-scar dataset as an independent hypertrophic-scar AFCO2 treatment-context comparator to test whether fixed keloid-derived programs showed similar or divergent responses in another pathological-scar context (25). Quantitative multiplex immunofluorescence and CD74 immunohistochemistry assessed tissue-level concordance for the principal epithelial and MHC-II-associated findings. The study was not designed to establish a causal molecular mechanism or a disease-type effect.

## Results

### Paired single-cell profiling resolves the cellular landscape of treated keloid scars

We jointly reprocessed eight patient-matched keloid specimens from four individuals. The four untreated PRE specimens were the hypercellular-zone samples previously reported in GSE335482, whereas the four POST specimens were newly generated 35 days after AFCO2 treatment (31). The previously published infiltrating-zone samples were excluded from the current analysis. All samples were collected using the same operational definition of the hypercellular zone and processed on the same single-cell platform. Sample-specific quality-control thresholds, broad cell-class counts and analysis units are summarized in **Supplementary Table S1**.

The integrated analysis object contained 82,127 cells and 37,493 genes. Prespecified quality-control, doublet and ambient-RNA filters retained 74,937 cells for the primary analysis, 71,130 for an ambient-sensitive analysis and 67,140 for a consensus-stringent analysis. The lower retained PRE cell count relative to the earlier regional study reflects the current doublet, ambient-RNA and consensus-stringent filtering framework rather than a change in sample identity. Fresh scDblFinder calls agreed with the historical classification for 90.9% of cells, and source-level differential-expression directions were highly concordant between the primary and stricter analysis sets (Spearman rho 0.985-0.996 across the three principal lineages; **Supplementary Figure S1** and **Supplementary Table S1**).

The primary atlas comprised 41,071 keratinocytes, 8,941 endothelial cells, 5,680 fibroblasts, 5,463 mural cells, 3,933 mast cells, 3,791 T cells, 1,855 myeloid/APC cells, 1,805 B cells, 1,048 Schwann/perineural-like cells, 699 melanocytes and 651 Langerhans cells (**Figures 1a–e**). Canonical marker expression and sample-contribution profiles supported the broad-source assignments. Cell recovery varied substantially across specimens, including 51,691 PRE and 23,246 POST cells; broad-source proportions were therefore treated as descriptive sample characteristics rather than cell-level treatment effects. A patient-condition keratinocyte pseudobulk heatmap provided a first view of paired epithelial remodeling (**Figure 1f**).

**Figure 1:**
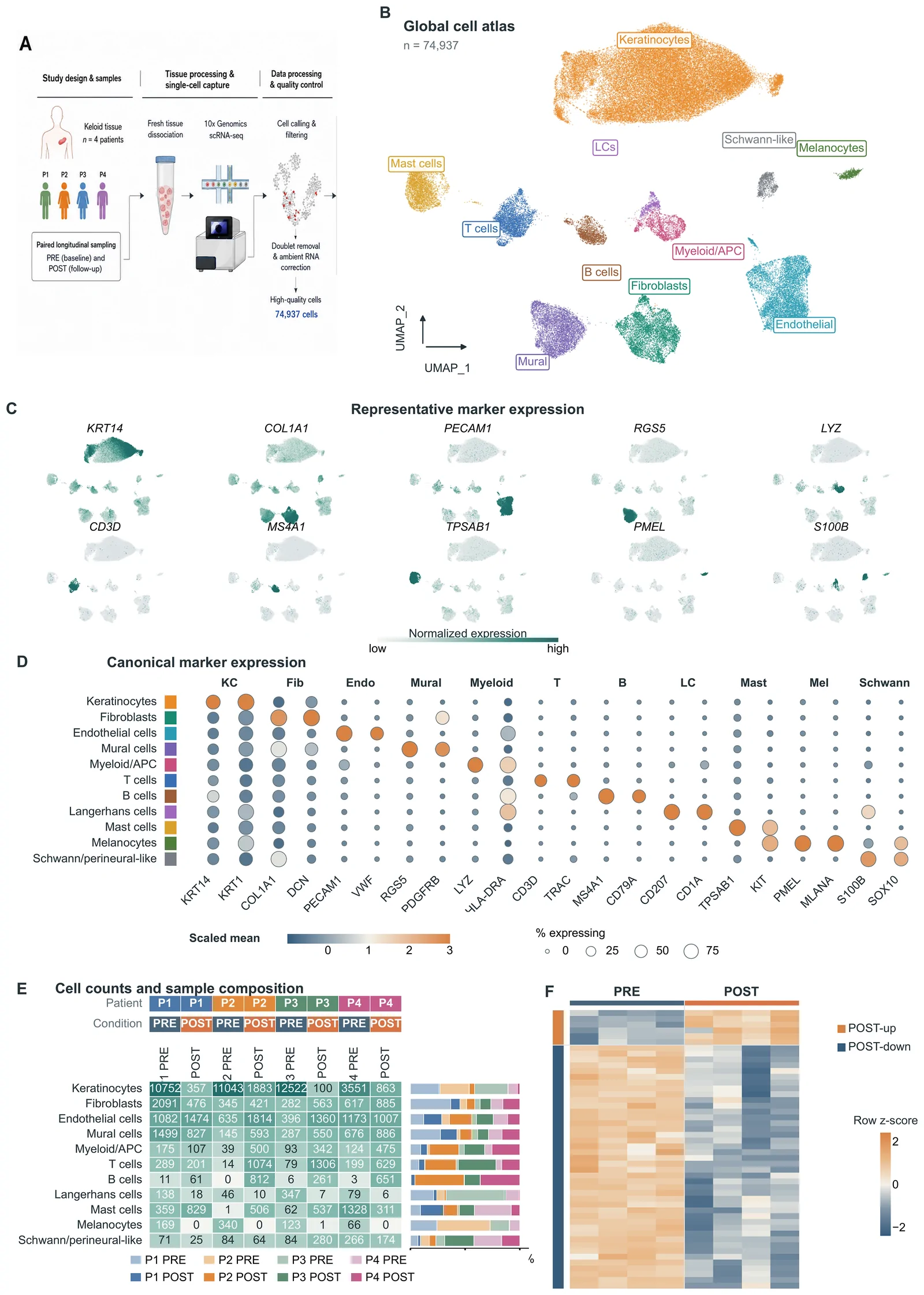
Paired study design and canonical cellular atlas. **(a)**, Study workflow showing previously reported untreated hypercellular-zone PRE samples from GSE335482, newly generated patient-matched POST samples obtained after AFCO2 treatment, Singleron Matrix/GEXSCOPE single-cell RNA sequencing, patient-aware analyses and tissue corroboration. The previously published infiltrating-zone samples were not included. **(b)**, UMAP of 74,937 primary-set cells annotated by broad source. **(c)**, Representative feature maps for canonical lineage markers; each map uses a gene-specific display range and is not directly comparable across genes. **(d)**, Canonical marker dotplot; color denotes scaled mean expression and dot size the percentage of expressing cells. **(e)**, Cell counts across the eight patient-condition samples and within-source sample composition. **(f)**, Patient-condition keratinocyte pseudobulk heatmap for the selected paired-expression landscape; values are row-scaled for visualization. Patient was the inferential unit for downstream treatment analyses; cell counts and UMAPs are descriptive.

Source-restricted analyses were performed for fibroblasts, myeloid/APC cells, endothelial/mural cells and keratinocytes. Fibroblast and endothelial/mural partitions were stable across graph, seed and leave-one-patient-out perturbations. Myeloid/APC partitions showed moderate leave-one-patient-out stability, whereas keratinocyte clustering was less stable. We therefore used descriptive state labels to localize transcriptional features, while prioritizing continuous programs and patient-level pseudobulk for treatment-associated inference.

### Fibroblast inflammatory reprogramming accompanies attenuation of myeloid antigen-presentation programs

Fibroblast subclustering resolved three principal descriptive states and pair-balanced consensus non-negative matrix factorization identified six continuous programs (**Figures 2a–d**). The interferon/inflammatory-remodeling program P1 increased in all four patients (mean POST-PRE usage delta +0.314). By contrast, the homeostatic ECM program P2 decreased in all patients (mean delta -0.255), and the ASPN/POSTN collagen-matrix program P3 decreased in three of four patients (mean delta -0.100). The remaining programs were retained in the complete response spectrum, but the three central programs captured the dominant paired pattern. Directional stability was preserved in leave-one-patient-out and ambient-filter sensitivity analyses (**Supplementary Figure S2**, **Supplementary Table S2**).

**Figure 2:**
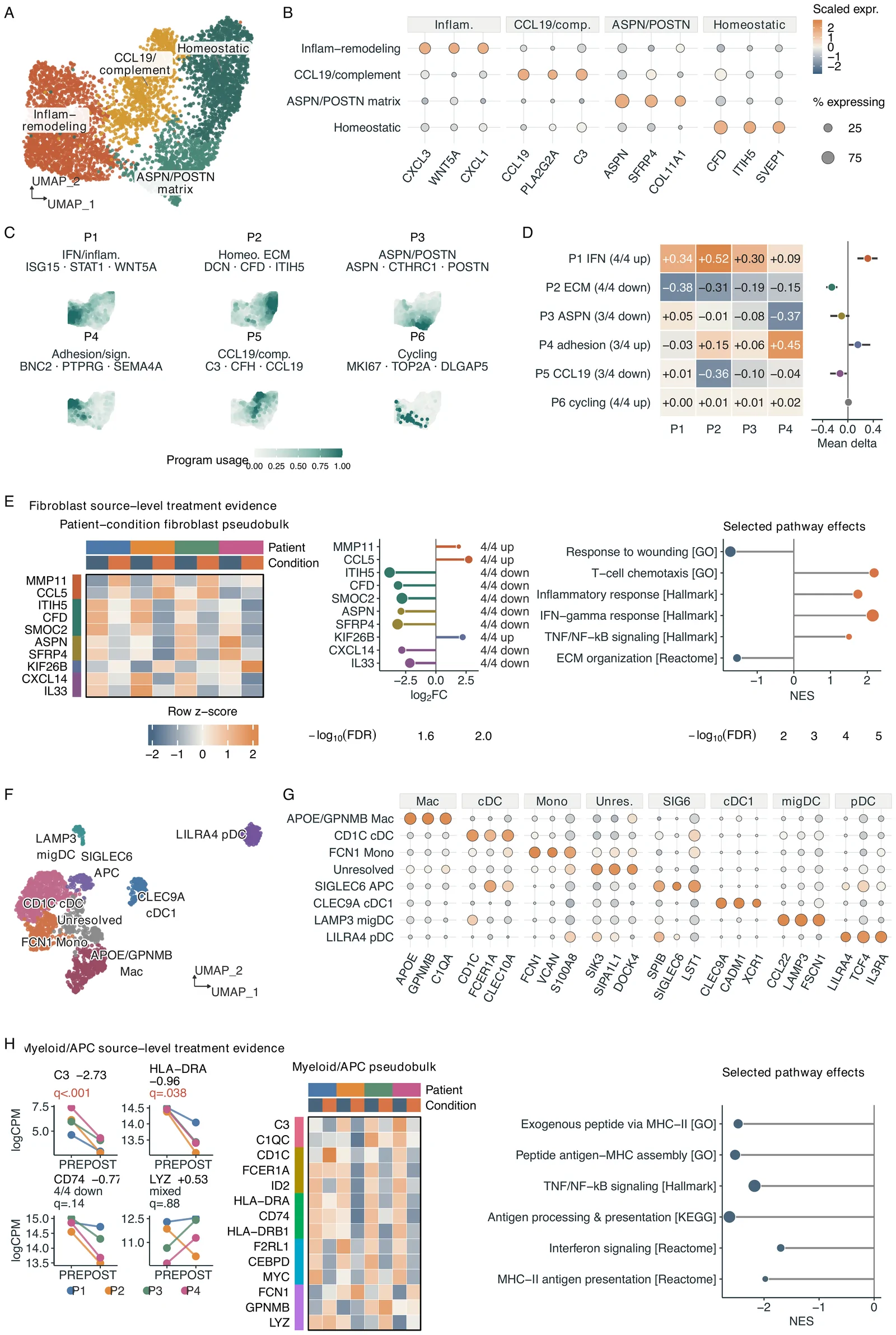
Fibroblast and myeloid remodeling after treatment. **(a)**, Fibroblast UMAP with three descriptive states. **(b)**, Marker expression across fibroblast states; color denotes scaled mean expression and dot size percentage expressing. **(c)**, Cell-level usage maps for six fibroblast cNMF programs. **(d)**, Patient-level POST-minus-PRE program changes, with patient values and summary effects. Treatment-associated contrasts were evaluated using four paired patients rather than individual cells as biological replicates. **(e)**, Fibroblast source-level evidence: patient-condition pseudobulk heatmap, selected paired gene effects and selected enrichment results. **(f)**, Myeloid/APC UMAP. **(g)**, Marker expression across myeloid/APC states. **(h)**, Myeloid/APC source-level evidence, including patient-level gene effects, pseudobulk expression and selected pathway enrichment. Pseudobulk models and program summaries used patients as replicates. State maps provide localization context and were not used as cell-level treatment tests.

Paired fibroblast pseudobulk supported loss of homeostatic and matrix-associated transcripts. SMOC2 (log2 fold change -2.81, FDR 0.00205), ITIH5 (-3.84, FDR 0.00307), CFD (-3.13, FDR 0.0125), ASPN (-2.89, FDR 0.0280) and COL14A1 (-1.57, FDR 0.0378) decreased in all four patients (**Figure 2e**). The paired ranking was positively enriched for interferon-gamma response (NES 2.15, FDR 6.07 × 10^-7) and TNF/NF-kB signaling (NES 1.50, FDR 0.0309), and negatively enriched for Reactome ECM organization (NES -1.55, FDR 0.0104). These results indicate a shift from homeostatic ECM programs toward an inflammatory fibroblast context; they do not directly measure matrix deposition, degradation or mechanics.

Myeloid/APC analysis identified a distinct paired response (**Figures 2f–h**). C3 decreased in all patients (log2 fold change -2.73, FDR 4.18 × 10^-4), as did HLA-DRA (-0.96, FDR 0.0384). CD74 was also lower in all four patients but did not meet the source-level FDR threshold (-0.77, FDR 0.140). LYZ was variable and nonsignificant (+0.53, FDR 0.881), precluding a source-level LYZ treatment claim. Antigen processing and presentation was negatively enriched in KEGG (NES -2.63, FDR 2.53 × 10^-7), GO MHC-II presentation (NES -2.47, FDR 8.94 × 10^-5) and Reactome MHC-II antigen presentation (NES -1.98, FDR 0.00202), alongside lower TNF/NF-kB signaling (NES -2.17, FDR 6.96 × 10^-8). The combined evidence supports attenuation of an antigen-presentation and inflammatory transcriptional context rather than uniform loss of myeloid cells.

### A KRT6-dominant epithelial program defines the principal matrix-interface response

Keratinocyte analysis resolved five descriptive states and six continuous cNMF programs (**Figures 3a–d**). The KRT6 injury-interface program P2, containing KRT6B, KRT6A, KRT16 and related injury-associated genes, increased in all four patients (mean usage delta +0.331). The AREG/NF-kB stress-regulatory program P1 decreased in all patients (mean delta -0.525), whereas the basal matrix-boundary program P3 was mixed. These continuous-program results were stable in leave-one-patient-out analyses and were given greater inferential weight than the less stable discrete keratinocyte partition.

**Figure 3:**
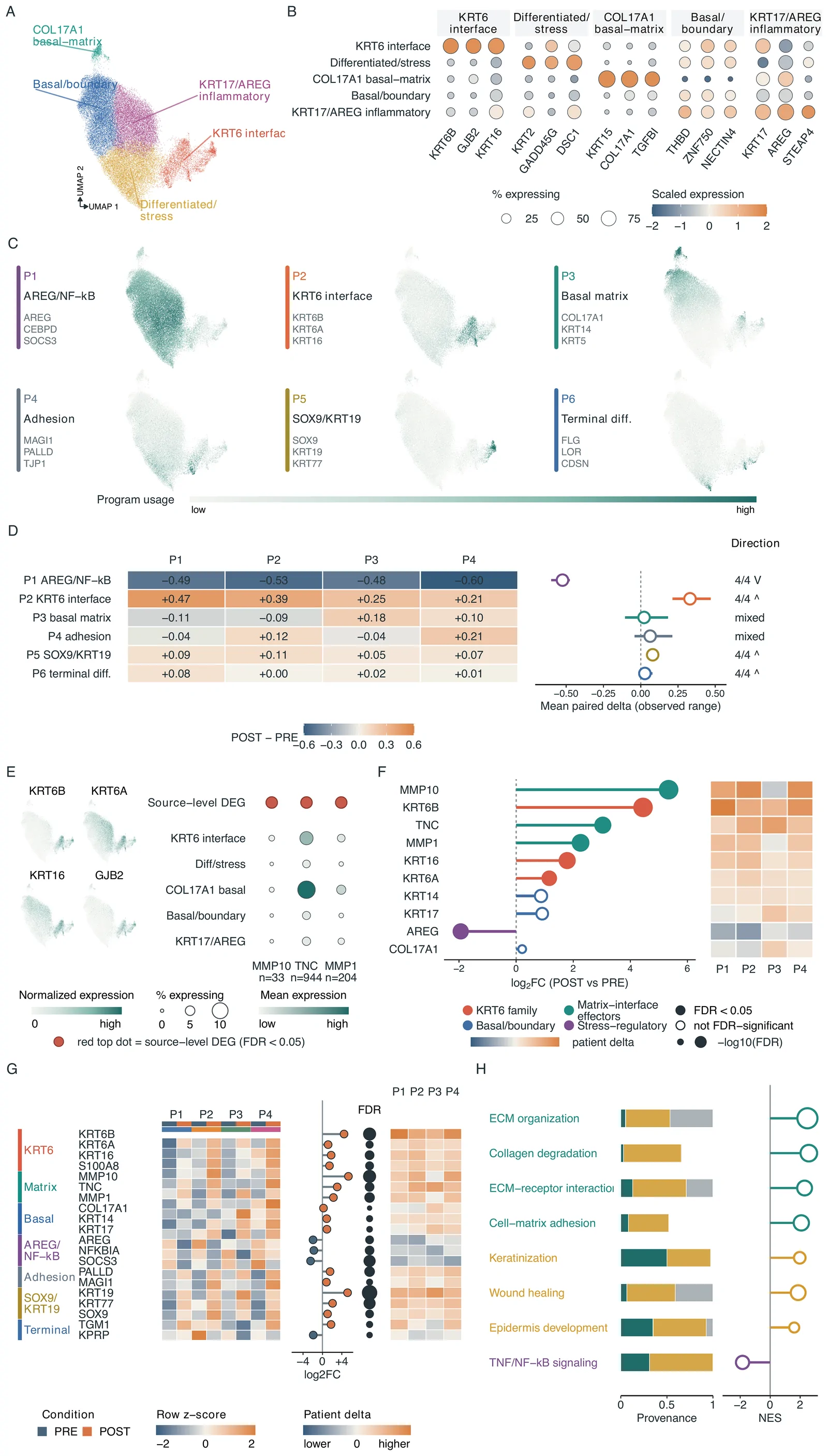
A KRT6-dominant epithelial program and matrix-interface transcriptional context. **(a)**, Keratinocyte UMAP with five descriptive states. **(b)**, State marker profiles; dot size denotes percentage expressing and color gene-wise scaled mean expression. **(c)**, Continuous usage maps for six keratinocyte cNMF programs. **(d)**, Patient-level POST-minus-PRE program changes and observed patient ranges. Treatment-associated contrasts were evaluated using four paired patients rather than individual cells as biological replicates. **(e)**, Feature maps for KRT6B, KRT6A, KRT16 and GJB2 together with state-level detection of MMP10, TNC and MMP1; red symbols denote source-level patient-aware FDR < 0.05 and not state-specific significance. **(f)**, Source-level keratinocyte pseudobulk effects with patient-specific direction matrix. **(g)**, Selected patient-condition keratinocyte pseudobulk heatmap, row-scaled for visualization. **(h)**, Provenance-filtered pathway summary; bars partition leading-edge genes by source-provenance class, and points show normalized enrichment score with size proportional to -log10(FDR). Complete unfiltered results and the source-provenance assessment are in **Supplementary Figure S2**.

Source-level paired pseudobulk resolved the epithelial response at gene level (**Figures 3e–g**). KRT6B increased by 4.44 log2 units (FDR 2.65 × 10^-5), KRT16 by 1.79 (FDR 0.00241), KRT6A by 1.16 (FDR 0.0217) and TNC by 3.04 (FDR 0.00248); each direction was positive in all four patients. MMP10 (+5.34, FDR 2.46 × 10^-5) and MMP1 (+2.26, FDR 8.13 × 10^-4) were significant but each had one discordant patient. AREG decreased in all patients (-1.94, FDR 0.00881). KRT17 showed a smaller, nonsignificant increase (+0.91, FDR 0.148) and was retained as a comparator rather than a co-primary marker. Sparse single-cell detection of MMP10 was displayed separately from source-level significance and was not used to imply broad expression in a discrete state.

The patient-aware keratinocyte ranking was positively enriched for Reactome ECM organization (NES 2.49, FDR 6.94 × 10^-16), collagen degradation (NES 2.58, FDR 5.93 × 10^-9), GO wound healing (NES 1.87, FDR 1.48 × 10^-6) and keratinization (NES 1.97, FDR 3.73 × 10^-4), whereas TNF/NF-kB signaling was negatively enriched (NES -1.84, FDR 4.78 × 10^-5; **Figure 3h**). A source-provenance assessment removed leading-edge genes with predominantly non-keratinocyte support from the main display without changing the underlying enrichment results; the complete source-provenance assessment and unfiltered results are reported in **Supplementary Figure S2** and **Supplementary Table S2**.

### The KRT6 program occupies a continuous epithelial context with model-dependent metabolic and regulatory features

DPT, Monocle3 and Slingshot each placed the KRT6-high state along a continuous epithelial ordering and showed a shift toward the KRT6-oriented endpoint in all four patients (**Figures 4a, b**). DPT and Monocle3 showed strong cell-order agreement (Spearman rho 0.796), whereas agreement with Slingshot was weaker. Program usage changed progressively along the ordered state space, and GeneTrajectory recovered an 89-gene ordered program that included KRT6B, KRT6A and KRT16 (**Figures 4c, d**). These analyses support an inferred epithelial-state continuum; they do not establish chronological progression, ancestry or lineage conversion.

**Figure 4:**
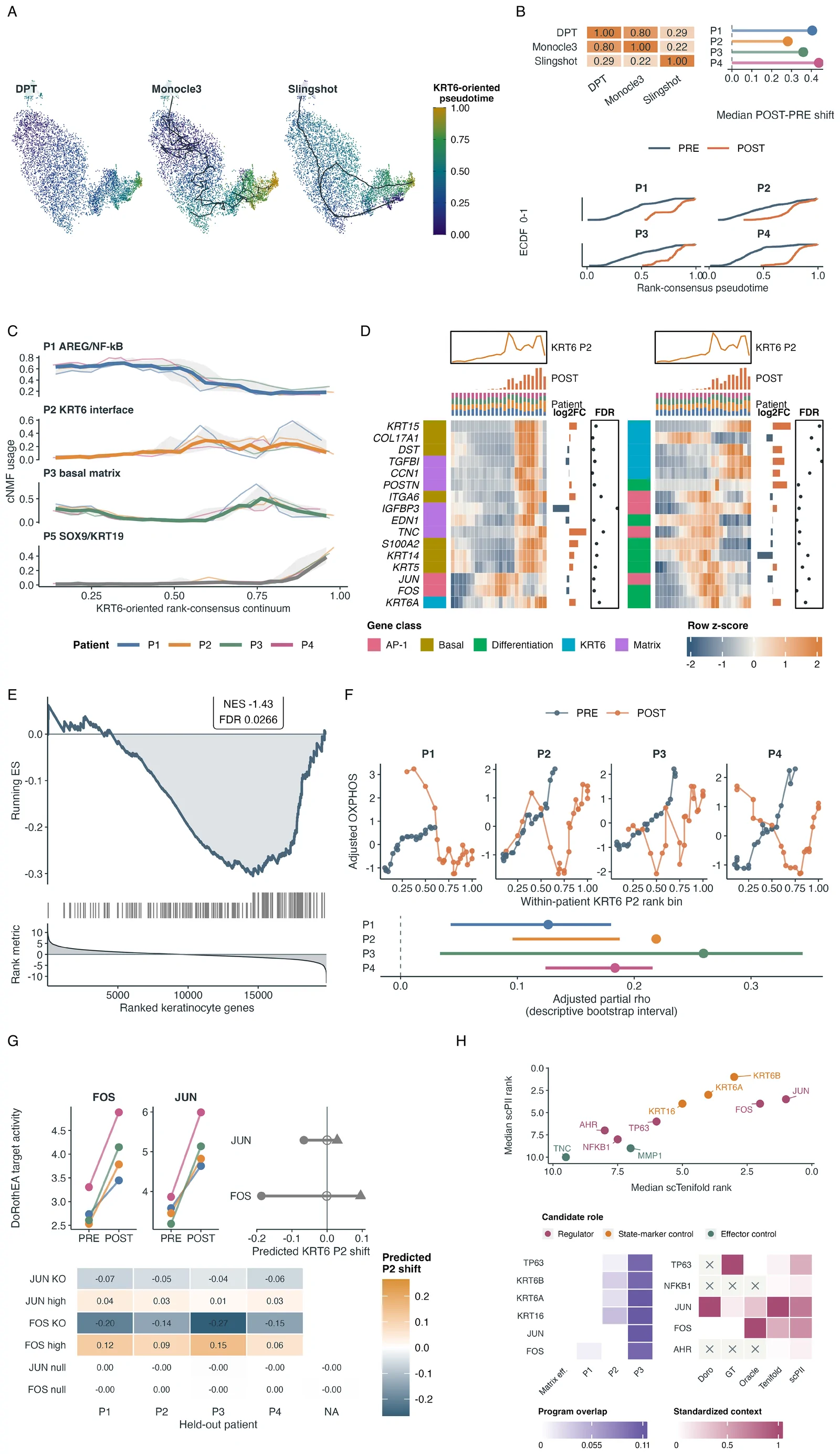
Dynamic, metabolic and computational context of the KRT6 epithelial program. **(a)**, KRT6-oriented cell ordering by diffusion pseudotime, Monocle3 and Slingshot. **(b)**, Method concordance, patient-level continuum shifts and PRE/POST empirical cumulative distributions. **(c)**, Patient-stratified cNMF program trends along the consensus epithelial continuum. **(d)**, GeneTrajectory-ordered heatmaps with program, condition and patient-contribution annotations. **(e)**, Preranked Hallmark oxidative-phosphorylation GSEA in keratinocyte pseudobulk. **(f)**, Within-patient adjusted association between KRT6 P2 and OXPHOS activity, with patient-specific estimates. **(g)**, FOS/JUN DoRothEA activity and CellOracle-predicted perturbation context. **(h)**, scTenifoldKnk/scPII expression-derived network context. Pseudotime is an inferred state ordering, not chronological treatment time; computational perturbations are model predictions and not functional experiments.

Hallmark oxidative phosphorylation was the only prespecified metabolic gene set that met the FDR criterion in the paired keratinocyte ranking (NES -1.43, FDR 0.0266; **Figure 4e**). After excluding genes overlapping KRT6 P2, within-patient adjusted associations between KRT6 P2 usage and OXPHOS activity were positive in all four patients but remained robust across the full sensitivity framework in three of four (**Figure 4f**). The apparently different treatment-level and within-state directions indicate scale-dependent transcriptional organization, not a uniform change in metabolic flux or dependency.

Sample-level DoRothEA activity increased for FOS and JUN in all four patients, and GeneTrajectory and CellOracle placed AP-1 factors within the KRT6-associated computational context (**Figure 4g**). Expression-derived network methods provided only partial support, and patient-held-out Geneformer predictions were directionally discordant (**Figure 4h**, **Supplementary Figure S2**). We therefore interpret AP-1 as a model-dependent hypothesis rather than an experimentally established upstream regulator.

### Global communication remodeling accompanies patient-consistent attenuation of mural-DC axes

Sample-wise CellChat analysis with the broad cell-class annotations provided architecture-level PRE and POST communication context (**Figures 5a, b**). Global networks were summarized using patient-supported median communication strength and were not interpreted as patient-level treatment effects. The circles, bubble matrix and source-pathway-receiver alluvial display describe redistribution of inferred architecture rather than physical contact or receptor activation.

**Figure 5:**
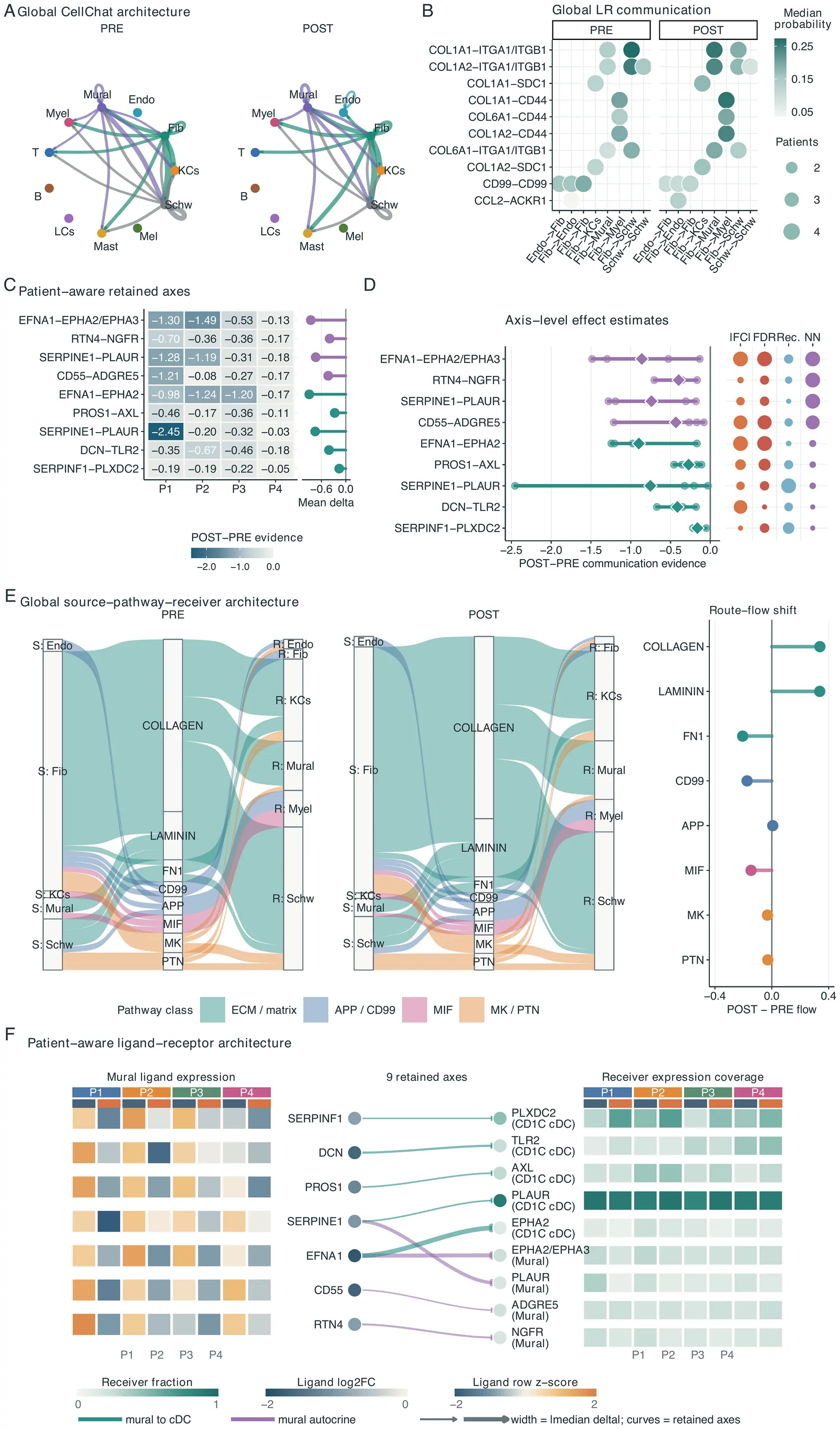
Global and patient-aware communication remodeling. **(a)**, PRE and POST global CellChat communication-strength circles using broad cell-class annotations. **(b)**, Global ligand-receptor bubble matrix; color denotes median communication probability and dot size patient support. **(c)**, Patient-specific POST-minus-PRE communication-evidence deltas for nine retained axes. Treatment-associated contrasts were evaluated using four paired patients rather than individual cells as biological replicates. **(d)**, Patient-level axis effects with aligned sender-ligand, receiver-expression and NicheNet evidence columns. **(e)**, Global source-pathway-receiver alluvial context and selected pathway information-flow summary. **(f)**, Focused ligand-receptor evidence butterfly for the nine retained mural-to-CD1C cDC-like and mural-autocrine axes. Curve width denotes the magnitude of the median patient-level evidence decrease; curves represent retained membership, not correlation or causal strength. Global CellChat panels are architecture-level context, whereas patient-level inference is restricted to the nine retained axes.

A separate patient-aware framework evaluated ligand-receptor axes using four-pair communication deltas, sender-ligand expression, receiver-receptor expression and NicheNet prior support (**Figures 5c–f**). Nine axes had complete evidence across all four patient pairs. Five linked mural/pericyte cells to CD1C conventional DC-like cells and four were mural autocrine axes. Every retained axis weakened in POST, and no strengthened axis met all prespecified evidence criteria.

The mural autocrine set comprised EFNA1-EPHA2/EPHA3, RTN4-NGFR, SERPINE1-PLAUR and CD55-ADGRE5. The mural-to-CD1C cDC-like set comprised EFNA1-EPHA2, PROS1-AXL, SERPINE1-PLAUR, DCN-TLR2 and SERPINF1-PLXDC2. Candidate axes involving TNC, FN1, AREG/HBEGF or MMP genes did not meet the prespecified criteria and are retained in **Supplementary Figure S3** and **Supplementary Table S3**. The focused result supports attenuation of inferred mural-DC and mural-autocrine communication context, not functional ligand-receptor validation.

### Independent hypertrophic-scar reprocessing reveals context-dependent laser responses

We independently reprocessed the D0 and W3 samples from GSE320017 rather than merging them with the keloid data (25). The high-confidence hypertrophic-scar atlas contained 44,240 cells and resolved three donor-robust fibroblast states (**Figures 6a, b**). The smaller keratinocyte compartment contained approximately 492 cells and only three eligible paired donors; it was therefore evaluated using fixed keloid-derived program projections rather than an equivalent *de novo* state hierarchy.

**Figure 6:**
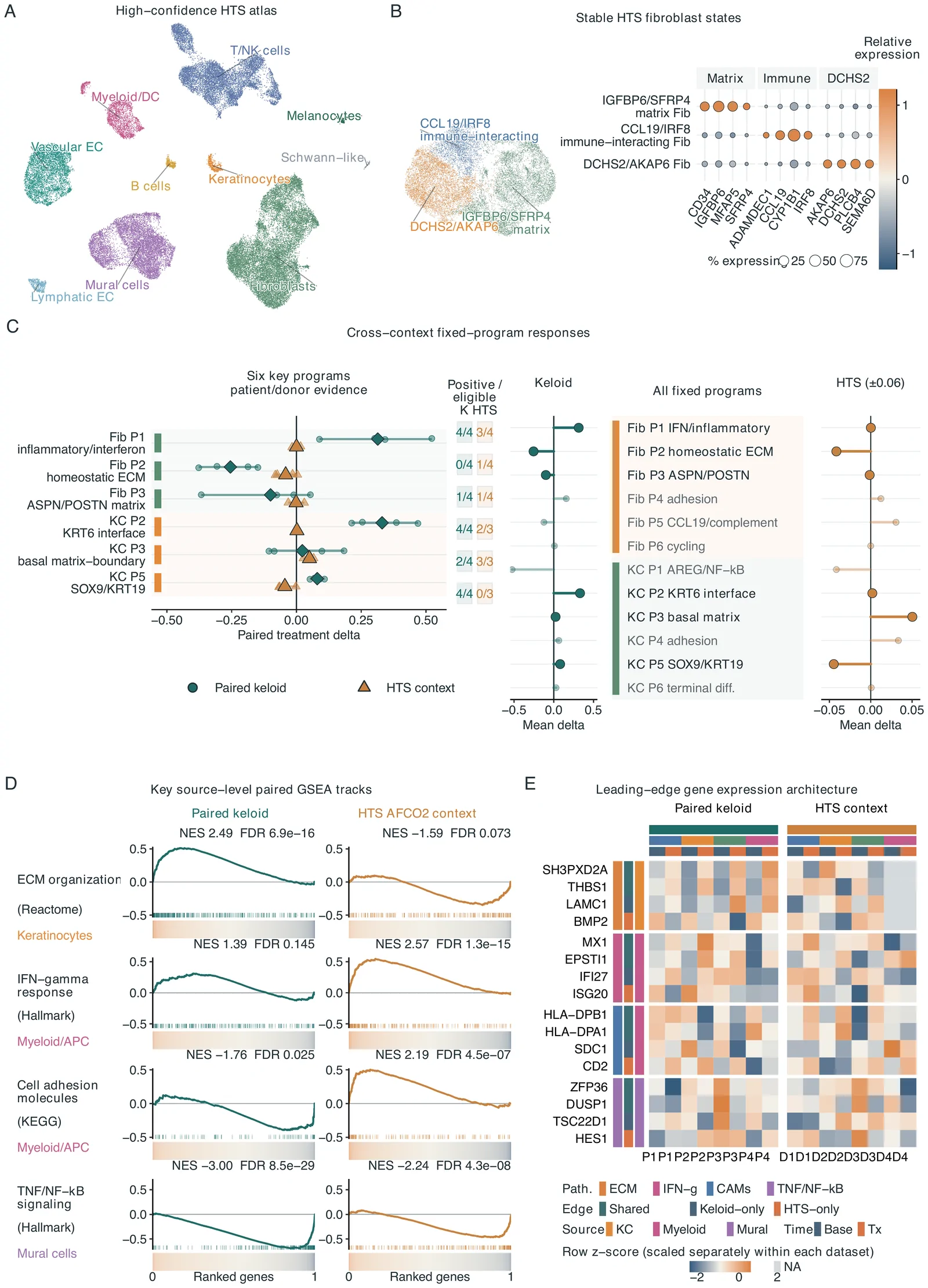
Independent hypertrophic-scar AFCO2 treatment-context comparison. **(a)**, High-confidence GSE320017 HTS atlas after independent reprocessing. **(b)**, Three donor-robust *de novo* fibroblast states and marker expression. **(c)**, Cross-context fixed-program responses: detailed patient/donor effects for six prespecified programs and the complete mean response spectrum for all 12 prespecified fibroblast and keratinocyte programs. Keloid and HTS mini-forests use independent display ranges (+/-0.55 and +/-0.06, respectively); segment lengths are not comparable across columns. **(d)**, Paired within-dataset running-GSEA tracks for four prespecified source-pathway contrasts; ranked universes were analyzed separately and no pooled cross-dataset test was performed. **(e)**, Sample-resolved expression of 16 deterministically selected leading-edge genes, row-scaled separately within each dataset; gray denotes source-ineligible or unavailable values. HTS is an independent treatment-context comparator, not external validation. The datasets differ in clinical composition, anatomical sampling, post-treatment interval and technical workflow. These comparisons describe within-dataset responses in different treatment contexts and do not establish an effect attributable specifically to scar type.

Patient- and donor-resolved estimates showed that the treatment-associated response was not uniform across scar contexts (**Figure 6c**). Keloid keratinocyte P2 increased by +0.331 with 4/4 patients positive, whereas the corresponding HTS estimate was +0.0019 with 2/3 donors positive. Keratinocyte P3 was mixed in keloid (+0.0226; 2/4 positive) but increased in 3/3 eligible HTS donors (+0.0503). Keratinocyte P5 increased in 4/4 keloid patients (+0.0803) but decreased in all three HTS donors (-0.0447). Fibroblast P1 showed a strong 4/4 increase in keloid (+0.314) but a near-zero, mixed HTS response, whereas fibroblast P2 decreased in both contexts but more strongly in keloid (-0.255 versus -0.0416). The complete 12-program spectrum was retained to avoid selective presentation.

Four source-level GSEA contrasts and a deterministic leading-edge heatmap provided pathway- and gene-level context (**Figures 6d, e**). Ranked universes and row scaling were kept separate within each dataset, and no pooled cross-dataset test or disease-type coefficient was estimated. The keloid and HTS mini-forests in **Figure 6c** use independent display ranges (+/-0.55 and +/-0.06, respectively); exact values are unchanged, and segment lengths are interpretable only within each column. Extended state, program and pathway results are provided in **Supplementary Figure S3** and **Supplementary Table S3**. GSE320017 therefore serves as an independent hypertrophic-scar AFCO2 treatment-context comparator, not external validation.

### Paired tissue imaging corroborates epithelial CK6-family remodeling and a reduced CD74-positive tissue context

We quantified the final multiplex immunofluorescence dataset across four paired patients, with five prespecified regions of interest per patient-condition treated as technical replicates. CK6-family-positive area within CK14-defined epithelium increased from a patient mean of 2.70% in PRE tissue to 65.84% in POST tissue (mean paired increase +63.13 percentage points; range +39.96 to +85.35; 4/4 patients; **Figures 7a, b**). MMP10-positive area overlapping CK14-defined epithelium increased from 8.49% to 25.92% (mean paired increase +17.43 percentage points; range +6.67 to +29.22; 4/4 patients). An exploratory CK6/CK14/MMP10 triple-overlap measure increased from 0.033% to 15.66% (mean paired increase +15.63 percentage points; range +11.98 to +20.82; 4/4 patients). For each endpoint, the two-sided exact sign-randomization P value was 0.125, the minimum attainable value for four non-zero concordant pairs, and was interpreted descriptively.

**Figure 7:**
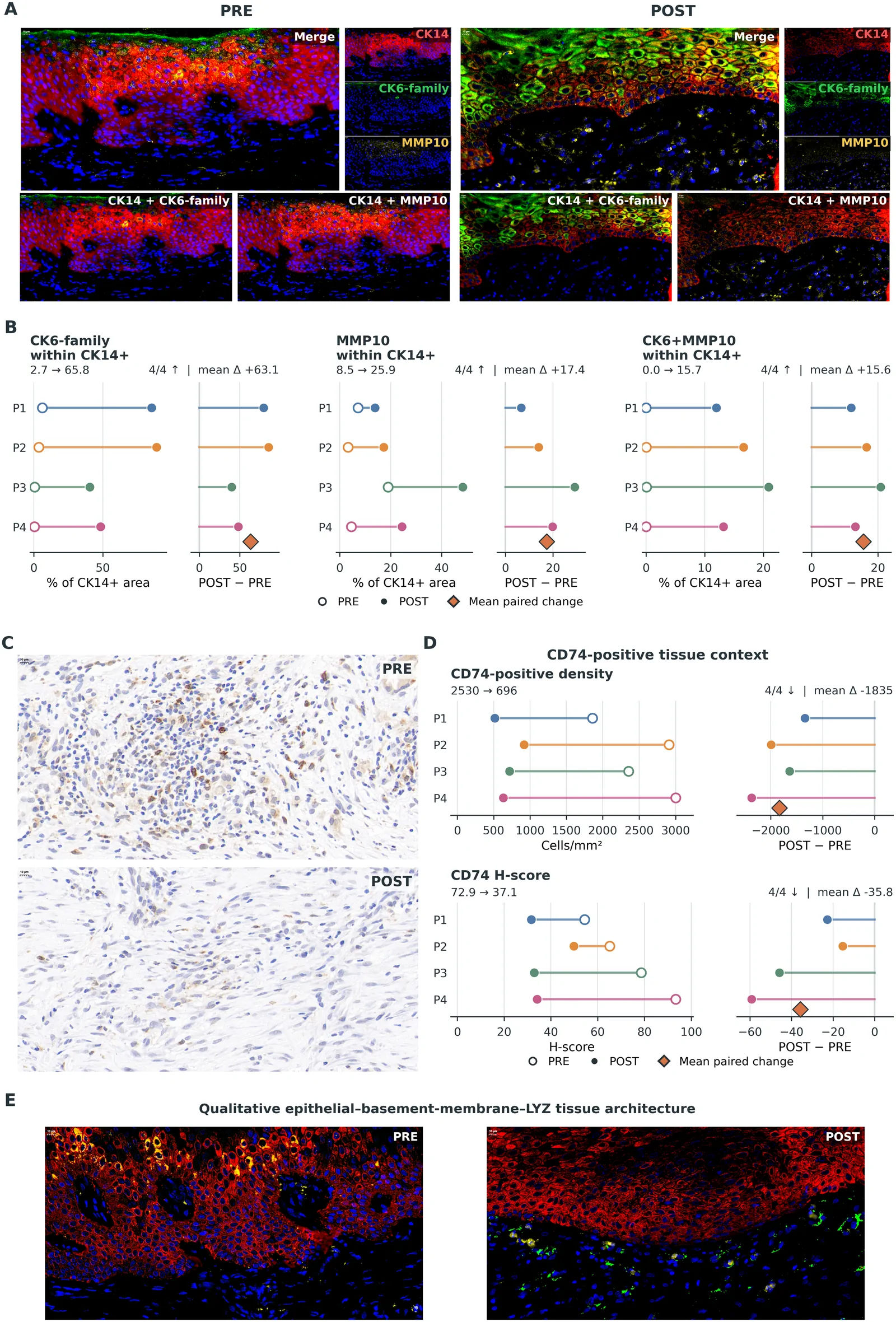
Paired tissue imaging of epithelial CK6-family/MMP10 and the CD74-positive tissue context. **(a)**, Matched-field PRE and POST multiplex immunofluorescence. Large merged images are accompanied by CK14 (red), CK6-family (green) and MMP10 (yellow/orange) single-channel views and dual-channel views. **(b)**, Patient-level CK6-family epithelial overlap, MMP10 epithelial overlap and exploratory CK6/CK14/MMP10 triple-overlap (Quantitative analyses included four paired patients. Five regions of interest were measured per patient-condition and treated as technical subsamples; the plotted PRE and POST values represent patient-level aggregates. Individual regions were not used as independent biological replicates.). Open and filled circles denote PRE and POST, right-side lollipops show POST minus PRE and diamonds the mean paired change. **(c)**, Representative PRE and POST CD74 immunohistochemistry. **(d)**, Patient-level CD74-positive nucleated-cell density and H-score. CD74 staining was interpreted as a tissue-level MHC-II-associated readout, not as a lineage-specific measure of antigen-presenting-cell abundance or a functional assay of antigen presentation. **(e)**, Qualitative PRE and POST DAPI/CK14/COL4/LYZ multiplex images illustrating epithelial, basement-membrane-associated and LYZ-positive tissue compartments. The older multiplex panel was not quantified. Regions were technical replicates and patient was the inferential unit; exact P values are reported in the source tables but not used as the principal visual message. Scale bars are shown in the images.

CD74 immunohistochemistry changed in the opposite direction (**Figures 7c, d**). Mean CD74-positive nucleated-cell density decreased from 2,530.35 cells/mm² in PRE tissue to 695.50 cells/mm² in POST tissue (mean paired difference -1,834.85 cells/mm²; range -2,368.60 to -1,341.40; 4/4 patients). Mean H-score decreased from 72.924 to 37.136 (mean paired difference -35.788; range -59.196 to -15.376; 4/4 patients). CD74-positive-cell percentage and positive-area percentage also declined in every patient. These findings provide tissue-level corroboration of a reduced MHC-II-associated context but do not identify the lineage or functional state of every CD74-positive cell (32).

The earlier CK14/COL4/LYZ multiplex images were retained as qualitative architecture context only (**Figure 7e**). They illustrate spatial juxtaposition of epithelial, basement-membrane-associated and LYZ-positive tissue compartments and were not used to claim cellular co-expression or a treatment effect. Wet-lab source data, endpoint definitions and input-integrity records are provided in **Supplementary Figure S4** and **Supplementary Table S4**.

## Discussion

This paired human study identifies coordinated epithelial, stromal, immune and communication remodeling in keloids after AFCO2 treatment. The most reproducible epithelial signal was a continuous KRT6-dominant program that increased in all four patients and was accompanied by KRT6B, KRT6A and KRT16 induction, ECM-remodeling enrichment and quantitative expansion of CK6-family signal within CK14-defined epithelium. Fibroblasts shifted away from homeostatic ECM programs toward an interferon/inflammatory context, whereas myeloid/APC cells showed lower MHC-II-associated and antigen-presentation programs. A stricter communication analysis identified nine mural-DC or mural-autocrine axes that weakened in every patient. These layers should be interpreted as a treatment-associated tissue response rather than as a single causal pathway.

The present study extends, rather than repeats, our previous regional atlas (31). That work compared untreated infiltrating and hypercellular zones and contained no post-laser specimens. Here, only the previously reported hypercellular-zone samples were reused as baseline data, all infiltrating-zone samples were excluded, and the new scientific question was the patient-matched PRE-to-POST response. The complete analytical workflow was rerun under the current quality-control and patient-aware framework.

The epithelial finding is biologically plausible in the context of wound repair. KRT6, KRT16 and KRT17 are rapidly induced in stressed and wound-proximal keratinocytes, where they modify cytoskeletal organization, adhesion, migration and barrier-associated signaling (27, 28). In our data, however, KRT17 was neither the largest epithelial effect nor FDR-significant, whereas KRT6B, KRT6A and KRT16 formed a patient-consistent continuous program. Quantitative mIF substantially strengthens this distinction: CK6-family area within CK14-defined epithelium increased in every patient, while CK14 area itself was mixed. The protein assay cannot distinguish KRT6A from KRT6B, so the tissue result is appropriately framed as CK6-family corroboration rather than KRT6B-specific validation.

MMP10 provides a complementary, but not mechanistically definitive, interface readout. Stromelysin-2/MMP10 is expressed by wound keratinocytes and can influence matrix processing and epithelial organization (29, 30). In the present study, MMP10 was significant at keratinocyte pseudobulk level and MMP10 overlap with CK14-defined epithelium increased in all patients. The triple-overlap measurement further localized CK6-family and MMP10 signals to the same epithelial region, but pixel-level overlap does not establish molecular interaction, regulation or a discrete triple-positive cell population. Functional perturbation will be required to determine whether MMP10 contributes to, follows or is independent of the KRT6 program.

The fibroblast response was not a simple increase in collagen production. Instead, continuous program analysis showed consistent inflammatory/interferon reprogramming and loss of homeostatic ECM context, supported by decreases in SMOC2, ITIH5, CFD, ASPN and COL14A1. This pattern is compatible with the broader view that fibroblast states in repair are dynamic and that matrix, immune and mechanical functions can be uncoupled (5–16). Recent mechanistic work in wound models has shown that fibroblast-state transitions can be governed by defined regulatory axes and can influence epithelial repair and vascular regeneration (26). Our paired human data do not reach that causal depth, but they indicate that epithelial remodeling occurred alongside a reproducible stromal-state shift rather than in isolation.

The myeloid findings extend this epithelial–stromal model. Significant reductions in HLA-DRA, C3 and MHC-II-associated gene sets, together with directionally concordant CD74 RNA and lower CD74-positive tissue density, support attenuation of an antigen-presentation context. Our previous regional study identified CD74-enriched fibroblasts in the untreated infiltrating margin relative to the hypercellular zone (31); that anatomical, fibroblast-focused contrast is distinct from the current treatment comparison and tissue-wide CD74 readout. CD74 is integral to MHC-II assembly and trafficking but also has functions beyond antigen presentation, including signaling as a macrophage migration inhibitory factor receptor (32). Moreover, single-marker IHC cannot distinguish dendritic cells, macrophages, Langerhans cells, B cells or inducible epithelial expression. The result should therefore not be interpreted as functional loss of antigen presentation or depletion of a defined APC population. Its value lies in cross-layer concordance with the broader myeloid transcriptional program.

Global and focused communication analyses addressed different questions. CellChat summarized architecture-level redistribution, whereas the nine retained axes required complete four-patient evidence, sender and receiver expression, and NicheNet support. Their uniform decrease suggests a selective attenuation of mural-DC and mural-autocrine communication context. Nevertheless, ligand-receptor inference remains dependent on expression, database priors and cell-state representation (49–51). It does not demonstrate physical contact, ligand availability, receptor activation or downstream function. The failed TNC, FN1, AREG/HBEGF and MMP candidates are therefore as important to the claim boundary as the retained axes: they prevent a visually appealing but unsupported epithelial-to-stromal mechanism from being promoted to the main conclusion.

Trajectory, metabolic and regulatory analyses add context rather than causality. The agreement between DPT and Monocle3 and the 4/4 patient shift toward the KRT6-oriented endpoint support continuous epithelial remodeling, but pseudotime is not chronological treatment time (41–43). Similarly, negative treatment-level OXPHOS enrichment coexisted with positive within-patient association between KRT6 usage and OXPHOS activity. This scale dependence argues against a uniform metabolic mechanism. DoRothEA, GeneTrajectory and CellOracle nominated AP-1-associated regulation, but other model families were not directionally concordant. These results are best used to prioritize future perturbation experiments rather than to infer that FOS/JUN or OXPHOS drives the tissue response (44–48, 52).

The hypertrophic-scar analysis was intended to assess whether fixed keloid-derived programs showed similar or divergent responses in another AFCO2-treated scar setting, rather than to validate the keloid findings in a matched external cohort. The source cohort differed from our all-female anterior-chest cohort in participant composition, anatomical sites and scar maturity. The follow-up intervals, laser settings, tissue handling and single-cell platforms also differed. Fitzpatrick phototype was reported for the source participants, but phototype matching and comparability of individual ultraviolet-exposure histories could not be established from the available metadata. Anatomical location alone was not treated as a reliable measure of sun exposure. Consequently, differences in program responses cannot be assigned specifically to scar type or to any single clinical characteristic. We estimated paired changes separately within each dataset and lineage, without pooling cells across studies or fitting a disease-type coefficient. The comparison provides treatment-context information while highlighting the need for clinically and technically matched cohorts to establish generalizability (25).

Several limitations should be considered. The biological sample size was four paired patients, all women with anterior-chest keloids. The findings therefore cannot be assumed to apply to men, keloids at other anatomical sites or more diverse patient populations. Anatomical site, local mechanical environment, sex and disease duration may contribute to differences in epithelial, stromal and immune responses, but their individual contributions could not be assessed in this cohort. There was no untreated longitudinal control, so treatment-associated changes cannot be separated fully from time, repeated biopsy or sampling-related effects. With four non-zero concordant patient pairs, the minimum attainable two-sided exact P value was 0.125; tissue-imaging results were therefore interpreted descriptively. Cell-class proportions were sensitive to tissue recovery, and protein-level measurements were limited to selected markers. CD74 immunohistochemistry did not resolve the lineage of positive cells, and the CK14/COL4/LYZ images were qualitative. The hypertrophic-scar comparison was not matched for all clinical or technical characteristics and was not used to estimate a disease-type effect. Computational trajectory, regulatory and communication findings remain hypotheses requiring functional testing.

Within these constraints, the study contributes a paired human view of keloid remodeling after laser treatment. The convergent increase in a KRT6-dominant epithelial program and CK6-family tissue signal, together with fibroblast-state redistribution, reduced MHC-II-associated context and altered communication architecture, supports a coordinated epithelial–stromal-immune response. These data provide a structured set of patient-consistent hypotheses for future studies using larger longitudinal cohorts, spatially resolved assays and direct perturbation of the KRT6/MMP10 and fibroblast-immune axes.

## Methods

### Study design and participants

This study analyzed four female patients aged 18–60 years with anterior-chest keloids. Each patient contributed one untreated hypercellular-zone PRE specimen and one patient-matched POST specimen obtained 35 days after AFCO2 treatment. The same clinical criteria were used to identify the hypercellular zone at both time points. The patient was the biological and inferential unit for all paired treatment analyses. Participants had received no local corticosteroid, 5-fluorouracil, laser, cryotherapy or radiotherapy before baseline sampling. The study was approved by the Ethics Committee of the Plastic Surgery Hospital, Chinese Academy of Medical Sciences and Peking Union Medical College (approval 2024-86). All participants provided written informed consent for study participation and publication of deidentified tissue images. Sample-specific quality-control thresholds, broad cell-class counts and analysis units are summarized in **Supplementary Table S1**.

The study was designed as a paired observational molecular analysis. No untreated longitudinal control was available. Accordingly, differences are described as treatment-associated or POST-versus-PRE changes rather than treatment-induced effects.

### Relationship to the previous publication and reuse of baseline data

The four PRE specimens correspond to the untreated hypercellular-zone samples previously reported in our regional single-cell study and deposited in GEO under accession GSE335482 (31). That study compared untreated infiltrating and hypercellular zones to investigate regional heterogeneity and contained no post-AFCO2 specimens. The present study adds four previously unpublished POST specimens from the same patients and addresses a different question: within-patient molecular and tissue changes after AFCO2 treatment. All previously analyzed infiltrating-zone samples were excluded, and the PRE and POST data were jointly reprocessed using the analytical framework described here. No previously published figure or table was reused, and the treatment-associated comparisons and tissue-imaging results are newly reported. Thus, this work extends the previously studied cohort with new longitudinal data rather than representing an independent patient cohort.

### Laser treatment and specimen collection

The 35-day interval was selected to coincide with the scheduled clinical follow-up and to provide a common post-treatment biopsy window for all four patients. This sampling time point was not used to assign the specimens to a discrete wound-healing phase or to assume that re-epithelialization or scar maturation had been completed. The analysis therefore characterizes the tissue state at this follow-up time point rather than the onset, peak or duration of the treatment response. All procedures were performed in an outpatient setting under local infiltration anaesthesia with 0.5% lidocaine, 0.25% ropivacaine and 0.01% epinephrine. Lesions were treated with an ablative fractional carbon dioxide laser (AFCO2; 10,600 nm) operated in ultrapulse mode, using a 0.2-mm spot size, 15 mJ and 1 W. POST biopsies were obtained from the same hypercellular region 35 days after treatment and before any additional intralesional or adjuvant therapy. Biopsies were divided for single-cell processing and formalin-fixed paraffin-embedded tissue analysis according to the prespecified specimen workflow shown in **Figure 1a**.

### Single-cell dissociation library preparation and sequencing

PRE and POST specimens were processed using the same protocol and platform. Fresh tissue was preserved in GEXSCOPE tissue-preservation solution and transferred to the laboratory under cold conditions. Samples were washed three times with Hanks balanced salt solution, minced into 1–2 mm fragments and digested in 2 ml GEXSCOPE tissue-dissociation solution (Singleron Biotechnologies, Nanjing, China). The suspension was filtered and centrifuged, and the pellet was resuspended in phosphate-buffered saline. Cell viability and concentration were assessed after trypan-blue and acridine-orange/propidium-iodide staining using a Countstar Rigel fluorescence cell counter.

Single-cell suspensions were adjusted to 2 x 10^5 cells ml^-1 and loaded onto microwell chips using the Singleron Matrix Single Cell Processing System. Libraries were prepared with GEXSCOPE Single Cell RNA Library Kits, pooled at 4 nM and sequenced on an Illumina NovaSeq 6000 with 150-bp paired-end reads. Reads were assessed with FastQC and fastp, adapters and poly(A) sequences were removed with cutadapt, and cleaned reads were aligned to GRCh38 with STAR. Gene and unique-molecular-identifier counts were generated with featureCounts. The PRE raw data were previously deposited in GSE335482; POST matrices were generated using the same workflow (31).

### Primary single-cell object and quality control

The integrated keloid analysis object contained 82,127 cells and 37,493 genes. Cell-level quality metrics, doublet detection and ambient-RNA sensitivity were prespecified without reference to the desired PRE/POST direction. Primary, ambient-sensitive and consensus-stringent analysis sets contained 74,937, 71,130 and 67,140 cells, respectively. scDblFinder was used for an independent doublet sensitivity analysis (35). Cell IDs, patient-condition identity and prespecified broad cell-class annotations were retained in metadata tables. Sample-specific upper cut-offs for detected genes/UMIs/mitochondrial fraction were P1 PRE, 4,835.330/19,662.547/24.203%; P1 POST, 5,889.236/17,133.119/28.305%; P2 PRE, 4,723.527/19,704.507/20.000%; P2 POST, 5,604.389/15,785.765/22.012%; P3 PRE, 4,279.158/13,892.954/25.757%; P3 POST, 5,166.131/13,662.028/20.016%; P4 PRE, 7,433.555/28,321.476/21.078%; and P4 POST, 7,170.685/20,313.468/30.227%. The integrated analysis object had already passed baseline quality control, and the data-driven lower gene and UMI bounds were zero in this sensitivity analysis. Extreme DecontX contamination was defined as the greater of 0.20 or the sample-specific 99th percentile (0.426-0.915).

### Broad atlas and lineage annotation

Normalization, variable-feature selection, dimensional reduction, neighbor-graph construction and UMAP visualization followed the prespecified single-cell analysis workflow (33, 34). Broad sources were assigned from coherent multi-gene marker sets and sample contribution rather than a single marker. The primary atlas included keratinocytes, fibroblasts, endothelial and mural cells, myeloid/APC cells, T cells, B cells, Langerhans cells, mast cells, melanocytes and Schwann/perineural-like cells. Source-restricted clustering was conducted for fibroblasts, myeloid/APC cells, endothelial/mural cells and keratinocytes. Stability was assessed across graph parameters, random seeds and leave-one-patient-out analyses. Descriptive state labels were applied after clustering and were not chosen to maximize treatment separation.

### Continuous transcriptional programs

Consensus non-negative matrix factorization was performed within eligible lineages using pair-balanced inputs (36). Program usage was treated as continuous. Patient-condition summaries were calculated before paired POST-PRE comparisons, and program identities were prespecified before figure rendering. Programs were not interpreted as discrete subtypes, cell populations or lineage transitions.

### Patient-aware pseudobulk differential expression

Raw counts were aggregated by patient, condition and broad source or eligible state. Paired models included patient and condition, with the exact implementation retained in the source-data tables. Log2 fold changes and Benjamini-Hochberg false-discovery rates were calculated at the biological-sample level. Patient-specific POST-PRE contrasts were used to report direction consistency. Pooled-cell P values were not used for treatment inference, consistent with recommendations for replicate-aware single-cell analysis (37). Differential expression was implemented with edgeR 3.40.2 using quasi-likelihood models.

### Gene-set enrichment and provenance filtering

Patient-aware ranked statistics were analyzed using preranked gene-set enrichment analysis with Hallmark, Gene Ontology, KEGG and Reactome collections (38, 39). Normalized enrichment scores and false-discovery rates were retained with database version and pathway identifiers. Leading-edge genes were traced to the prespecified ranked gene list. Keratinocyte genes used in matrix-interface displays were assessed for broad-source provenance, sample coverage, patient dominance and ambient/doublet sensitivity. Genes predominantly supported by other sources were removed from the main display but not from the underlying enrichment result. Heatmaps were rendered with fixed sample order using ComplexHeatmap (40).

### Pseudotime and ordered-gene analyses

Diffusion pseudotime, Monocle3 and Slingshot were applied to the prespecified patient-balanced keratinocyte dynamics set (41–43). Root orientation was defined from the basal/KRT6-low context and did not use PRE/POST labels. Method-specific pseudotime values were projected onto the canonical keratinocyte UMAP for visual comparison; projected line length, curvature and local proximity were not interpreted. GeneTrajectory was used to identify ordered transcriptional programs (44). Pseudotime was treated as a state-ordering variable, not chronological time or ancestry.

### Metabolic and regulatory context

Prespecified metabolic gene sets were tested by paired source-level GSEA. Cell-level pathway scores were analyzed separately from source-level enrichment. Genes overlapping the KRT6 P2 program were removed before within-patient KRT6-OXPHOS association analysis, and scores were adjusted within patient for technical covariates and condition. DoRothEA activity was estimated from patient-condition pseudobulks (45). CellOracle, scTenifoldKnk and patient-held-out Geneformer were analyzed as separate model families (46–48). Leave-one-patient-out and randomized-network checks were retained where available. All perturbation outputs were interpreted as model-dependent predictions rather than experimental knockout, overexpression or causal effects.

### Cell-cell communication analysis

CellChat was applied with the broad cell-class annotations to summarize PRE and POST communication architecture (49). Sample-wise outputs were aligned and summarized using patient-supported median communication strength. A separate focused analysis integrated patient-specific LIANA/consensus deltas, sender-ligand pseudobulk expression, receiver-receptor expression and NicheNet prior support (50, 51). Nine axes with complete evidence in all four patient pairs met all prespecified evidence criteria. Ligand-receptor arcs denote retained membership and evidence context; they do not represent correlation, physical interaction or causal signaling.

### Independent reprocessing of GSE320017

GSE320017 was selected because it provides longitudinal human hypertrophic-burn-scar samples before and after AFCO2 treatment (25). Its single-cell subset comprised four donors from a 14-participant clinical study; their characteristics are summarized in **Supplementary Table S3**. The original protocol included three treatments at three-month intervals and 5-mm biopsies at baseline, week 3, month 6 and year 1. We restricted the comparison to D0/W3 pairs, representing the earliest available follow-up before subsequent laser sessions. W3 corresponds to 21 days, whereas our keloid POST specimens were collected at 35 days. The comparator also differed in tissue handling and platform, using cryopreserved biopsies and 10x Chromium 3′ v3.1 rather than our fresh-tissue Singleron/GEXSCOPE workflow. Reported laser settings included DeepFX at 17.5–20 mJ and 5% density and/or SCAARFX at 110–150 mJ and 3% density. These differences were considered when interpreting cross-dataset findings. GSE320017 was reprocessed independently by sample as an independent hypertrophic-scar AFCO2 treatment-context comparator (25). D0 and W3 were used for the primary quantitative analysis. Broad-cell clustering and source-restricted analyses were marker-blinded during graph construction, and stability was assessed across resolution, seed, donor contribution and leave-one-donor-out recovery. An ambiguous one-sample-dominated cluster was excluded from the main high-confidence atlas, leaving 44,240 cells. Three fibroblast states met the main-figure stability criteria. The smaller HTS keratinocyte compartment contained approximately 492 cells and three eligible donor pairs and was evaluated using fixed keloid-derived program projections rather than an equivalent state hierarchy. Keloid POST-PRE and HTS W3-D0 effects were estimated separately within each dataset and lineage. No pooled cross-dataset significance test or disease-type coefficient was calculated.

### Multiplex immunofluorescence and immunohistochemistry

Paraffin-embedded keloid tissue sections were deparaffinized and rehydrated, followed by antigen retrieval in EDTA buffer (pH 8.0) at 70 °C for 4 h. Endogenous peroxidase activity was quenched with 3% hydrogen peroxide in methanol for approximately 25 min at room temperature in the dark. Sections were then blocked with 3% bovine serum albumin for 30 min at room temperature.

Two sequential three-marker multiplex immunofluorescence panels were used. All primary antibodies were obtained from Servicebio (Wuhan, China). The principal panel was stained in the order CK6-family (mouse; GB12824; 1:5000), CK14 (mouse; GB150086; 1:10000) and MMP10 (rabbit; GB112994; 1:10000). The qualitative panel was stained in the order collagen IV (rabbit; GB155688; 1:5000), CK14 (mouse; GB150086; 1:10000) and lysozyme (mouse; GB152948; 1:10000). Each primary antibody was incubated overnight at 4 °C in a humidified chamber.

Each staining cycle used the corresponding ready-to-use S-vision HRP-polymer detection reagent (goat anti-mouse, G1301; goat anti-rabbit, G1302; Servicebio) for 50 min at room temperature, followed by tyramide signal amplification for 10 min at room temperature in the dark. The first, second and third targets in each panel were detected using iF488-Tyramide (G1231), iF555-Tyramide (G1233) and iF647-Tyramide (G1232), respectively, each diluted 1:500. Between staining cycles, antibody elution was performed using G1266 elution solution for 5 min at room temperature, followed by replacement with fresh solution and incubation at 37 °C for 30 min. Sections were washed and blocked again before application of the next primary antibody. Nuclei were counterstained with DAPI (G1012) for 10 min at room temperature in the dark. Tissue autofluorescence was quenched using G1221 for 5 min, followed by washing and mounting with an antifade medium (G1401). All detection and mounting reagents were obtained from Servicebio. Protein-level CK6 findings are reported as CK6-family immunoreactivity rather than isoform-specific KRT6A or KRT6B signals. The CK14/COL4/LYZ images were retained for qualitative tissue architecture only.

For CD74 immunohistochemistry, keloid tissue was fixed in 4% paraformaldehyde, dehydrated through graded alcohols and embedded in paraffin. Sections were deparaffinized, rehydrated and subjected to antigen retrieval. Endogenous peroxidase activity was quenched with hydrogen peroxide, and sections were incubated overnight at 4 °C with an anti-CD74 primary antibody (Servicebio, GB115427; 1:500). After washing, an appropriate HRP-conjugated secondary antibody was applied, and staining was developed using 3,3′-diaminobenzidine. Sections were counterstained with haematoxylin, dehydrated, cleared and mounted with neutral resin for microscopic examination and image acquisition. PRE and POST sections within each assay were processed and imaged using the same batch-specific settings.

Five prespecified 600 x 300 micrometer regions of interest centered on the dermal-epidermal interface were analyzed per patient-condition for the CK6-family/CK14/MMP10 and CD74 assays. Regions were technical replicates; the patient was the inferential unit. Positive-area or positive-cell thresholds were defined within each assay and applied unchanged to PRE and POST images using AIpathwell/SaiViewer (Servicebio). The older CK14/COL4/LYZ images were not included in quantitative source data.

### Tissue-image quantification

For multiplex immunofluorescence, raw positive areas were summed across the five regions before ratios were calculated. The primary endpoint was 100 x sum(CK6/CK14 colocalized positive area)/sum(CK14-positive area). The key secondary endpoint replaced CK6/CK14 with MMP10/CK14 colocalized area. The exploratory endpoint used CK6/CK14/MMP10 triple-overlap area relative to CK14-positive area. Whole-field CK6-family, MMP10 and CK14 positive-area percentages were calculated from summed positive and tissue areas rather than by averaging regional percentages.

For CD74 IHC, the primary endpoint was positive nucleated-cell density weighted by analyzed tissue-pixel area. The final export did not include total cell counts; CD74-positive-cell percentage and H-score were therefore summarized as unweighted means across five equal-area regions, and this limitation was retained. Positive-area percentage was recalculated from summed positive-area and tissue-area pixels.

### Statistical analysis

For all PRE–POST treatment comparisons, the patient was the biological replicate and inferential unit. Individual cells contributed to cell-state characterization and within-sample summaries but were not treated as independent patient replicates. For tissue quantification, the five regions of interest per patient-condition were technical subsamples of the same specimen, and their measurements were aggregated before patient-level paired comparisons. Donors were treated analogously in the independent hypertrophic-scar analysis. Paired differences were defined as POST minus PRE. For every patient-level endpoint we report PRE, POST, paired difference, mean and median paired differences, observed range and direction count. A two-sided exact sign-randomization test enumerated all 2^4 patient sign assignments using the absolute mean paired difference as the statistic. With four non-zero concordant pairs, the minimum attainable two-sided P value was 0.125; these P values were therefore treated descriptively. No ROI-level inference was performed. False-discovery rates for transcriptomic analyses were taken from the prespecified replicate-aware models. Missing patient, donor, sample or gene values were not imputed for a main inferential result.

### Software and reproducibility

Each manuscript panel has a source-data registry and rendering script. **Figure 7** quantification and rendering used Python 3.9.6, pandas 2.3.3, SciPy 1.13.1, Matplotlib 3.9.4, Pillow 11.3.0 and openpyxl 3.1.5. **Figures 1**-**6** were generated with Python 3.9.6 and R 4.2.3; the formal CellChat analysis used R 4.4.3. Verified major packages were anndata 0.10.8, NumPy 2.0.2, pandas 2.3.3, SciPy 1.13.1, cNMF 1.7.1, Seurat 5.0.1, scDblFinder 1.12.0, harmony 1.2.0, edgeR 3.40.2, limma 3.54.2, CellChat 1.6.1 and ComplexHeatmap 2.22.0. The OSF reproducibility package contains the final code, relative-path configuration files, processed manuscript-facing data and software-environment records.

## Code availability

Custom analysis and figure-rendering code, relative-path configuration files, software-environment records and reproduction instructions are available through the same private anonymized OSF view-only link: https://osf.io/q83zw/overview?view_only=09393f2be86f44369f1f29571b4c0c65. The code package excludes raw sequencing data, patient-identifying information and proprietary software. A public OSF release will be created on publication.

## Data Availability

The datasets presented in this study can be found in online repositories. The names of the repository/repositories and accession number(s) can be found in the article/**Supplementary Material**.
