# Supplementary material for: Paired single-cell profiling reveals epithelial–stromal remodeling after ablative fractional carbon dioxide laser treatment in keloids
Source: Front Immunol. 2026 Oct 28;17:1992388. doi: 10.3389/fimmu.2026.1992388 (PMC13751414; doi:10.3389/fimmu.2026.1992388)
Supplement: Supplementary file 1 [file DataSheet1.pdf]

Supplementary Information

Paired single-cell profiling reveals epithelial–stromal remodelling after ablative fractional carbon dioxide laser treatment in keloids

Authors

Dongxian Lin, Li Duan, Bincheng Wang, Xueying Chen, Yuchen Cao, Yuanyuan Xu, Shanbaga Zhao and Lianzhao Wang

Supplementary figures and tables

Supplementary Figures S1–S4 present quality-control, sensitivity, communication, treatment-context and tissue-quantification evidence. Supplementary Tables S1–S4 provide the corresponding manuscript-facing source data.

Supplementary Figure S1

Supplementary Figure S1 | Quality control, sample contribution and lineage robustness. a, Cell numbers retained in the primary, ambient-sensitive and consensus-stringent analysis sets; sample-specific upper thresholds for detected genes, unique molecular identifiers, mitochondrial transcript fractions and ambient-RNA scores; and broad cell-class contributions across patient-condition samples. b, Patient-condition representation and within-lineage fractions for fibroblast and myeloid/APC states, with total cell counts and observed ranges.

Supplementary Figure S1a | Quality control and sample contribution

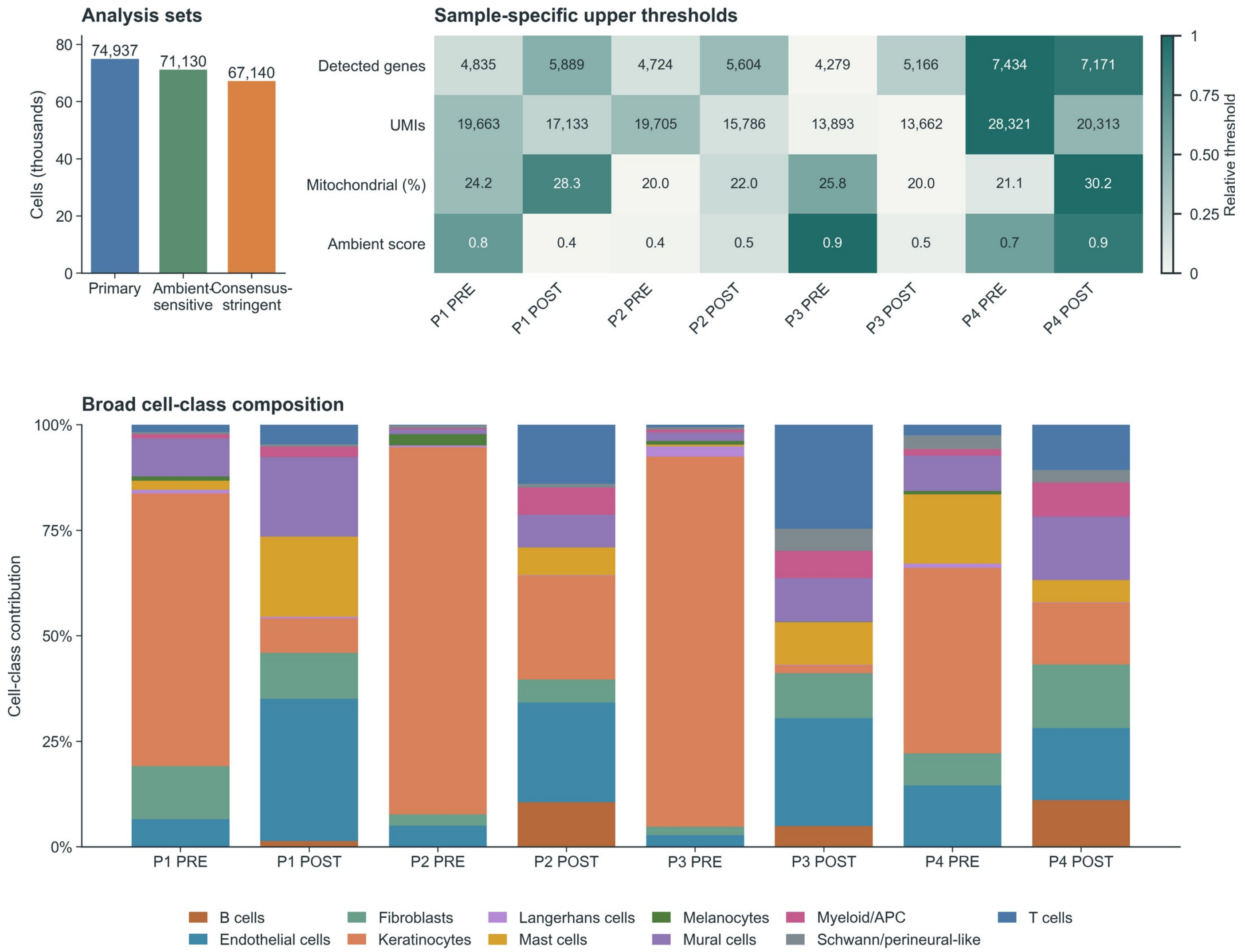

Supplementary Figure S1b | State representation and robustness

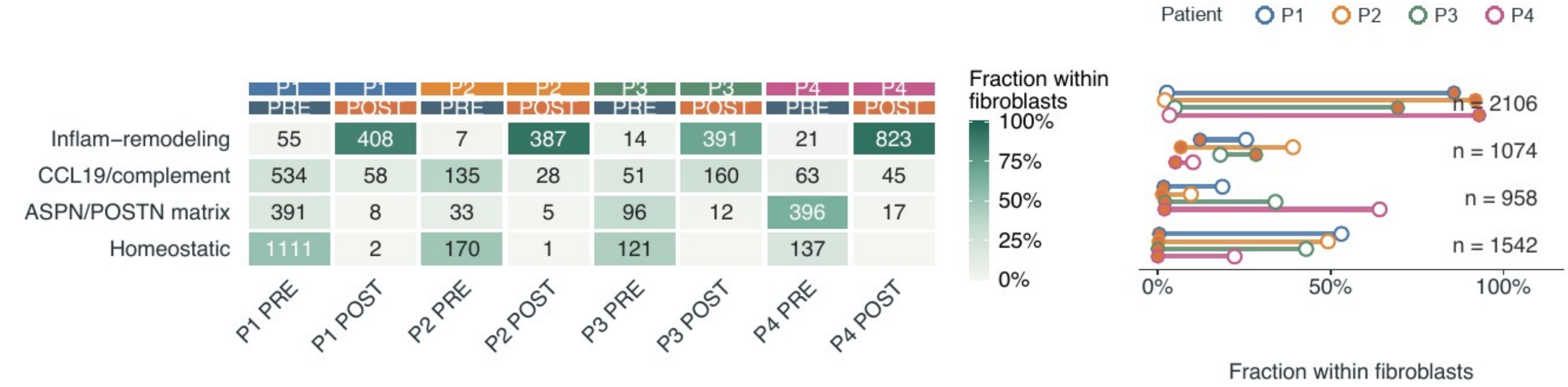

B

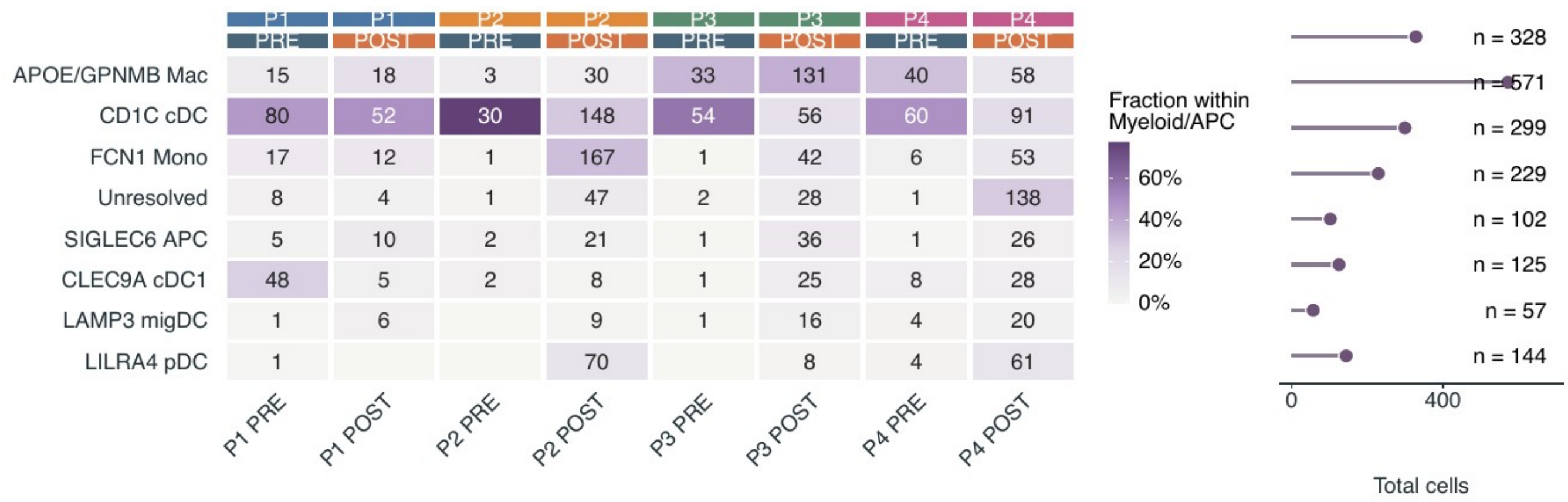

Supplementary Figure S2

Supplementary Figure S2 | Source-level transcriptional, trajectory and regulatory context. a, Paired source-level pseudobulk and enrichment results for fibroblasts and myeloid/APC cells. b, Running-enrichment profiles for four prespecified keratinocyte pathways, with ranked metrics, gene-hit rugs, normalized enrichment scores and false-discovery rates. c, Keratinocyte source-provenance assessment for genes with limited or non-keratinocyte support, including leave-one-patient-out direction stability. d, Metabolic pathway and reaction summaries, held-out-patient perturbation analyses and cross-method network context.

Supplementary Figure S2a | Complete source-level evidence

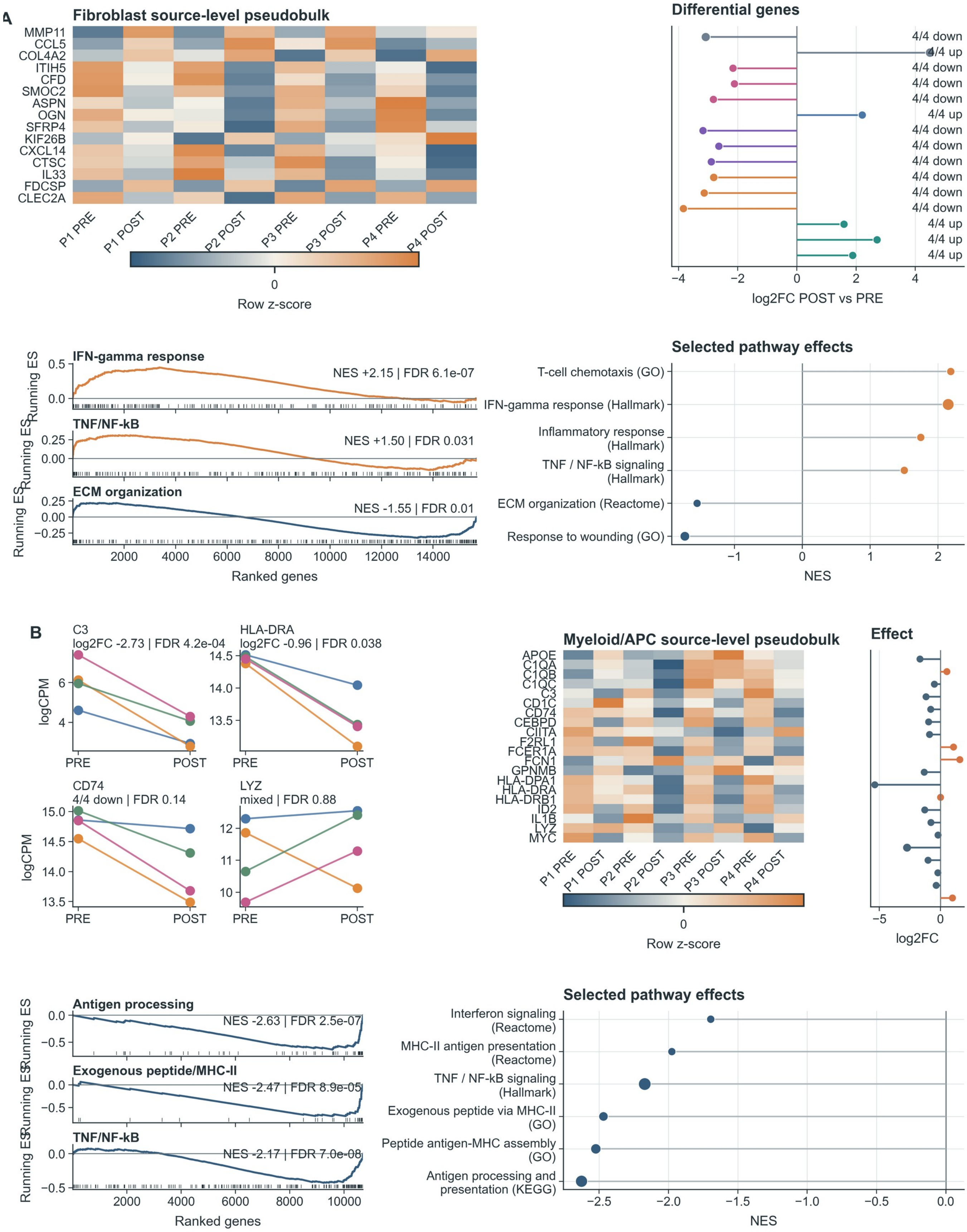

Supplementary Figure S2b | Selected keratinocyte running-GSEA profiles

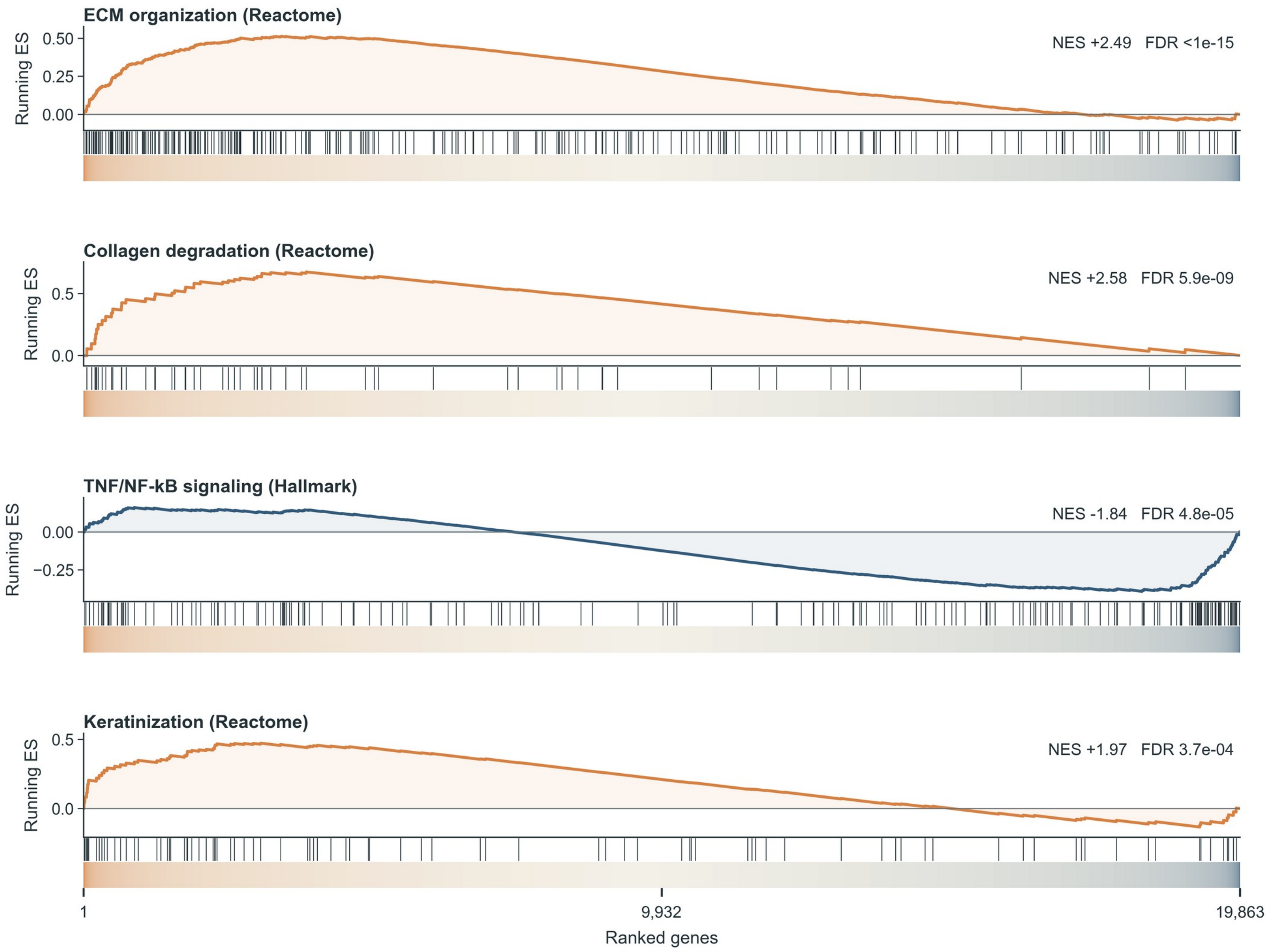

Supplementary Figure S2c | Keratinocyte source-provenance assessment

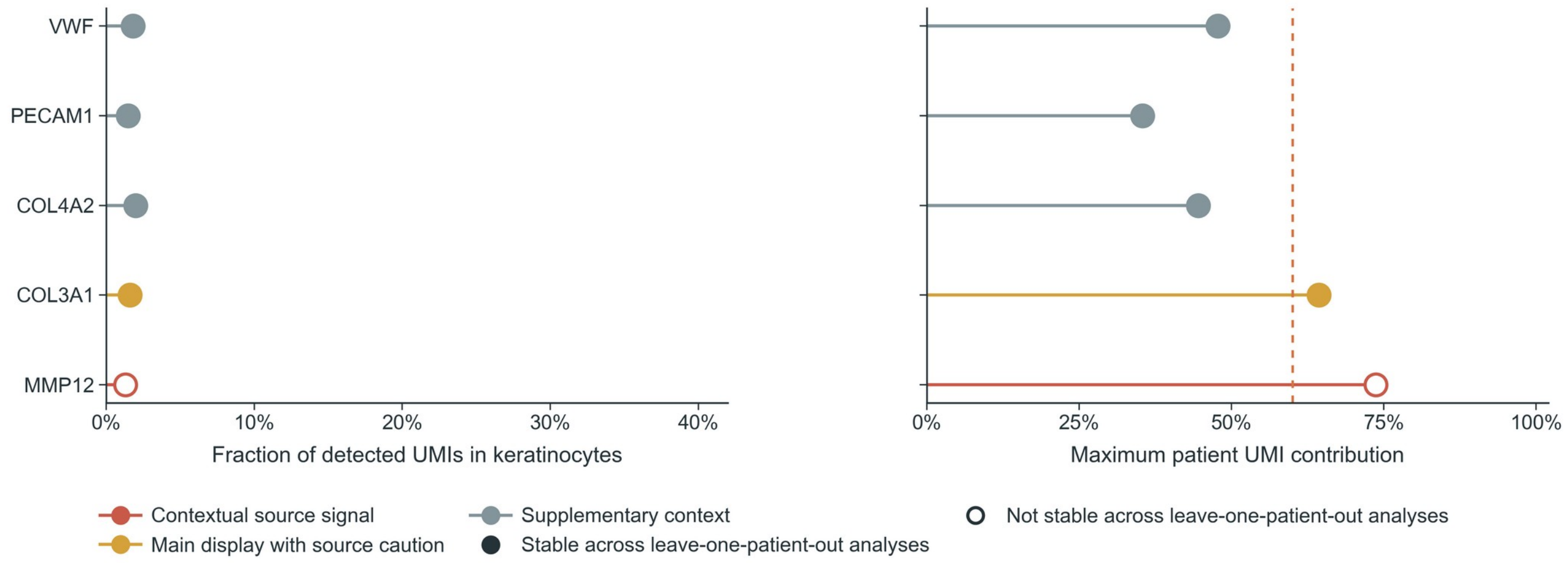

Supplementary Figure S2d | Trajectory and regulatory sensitivity

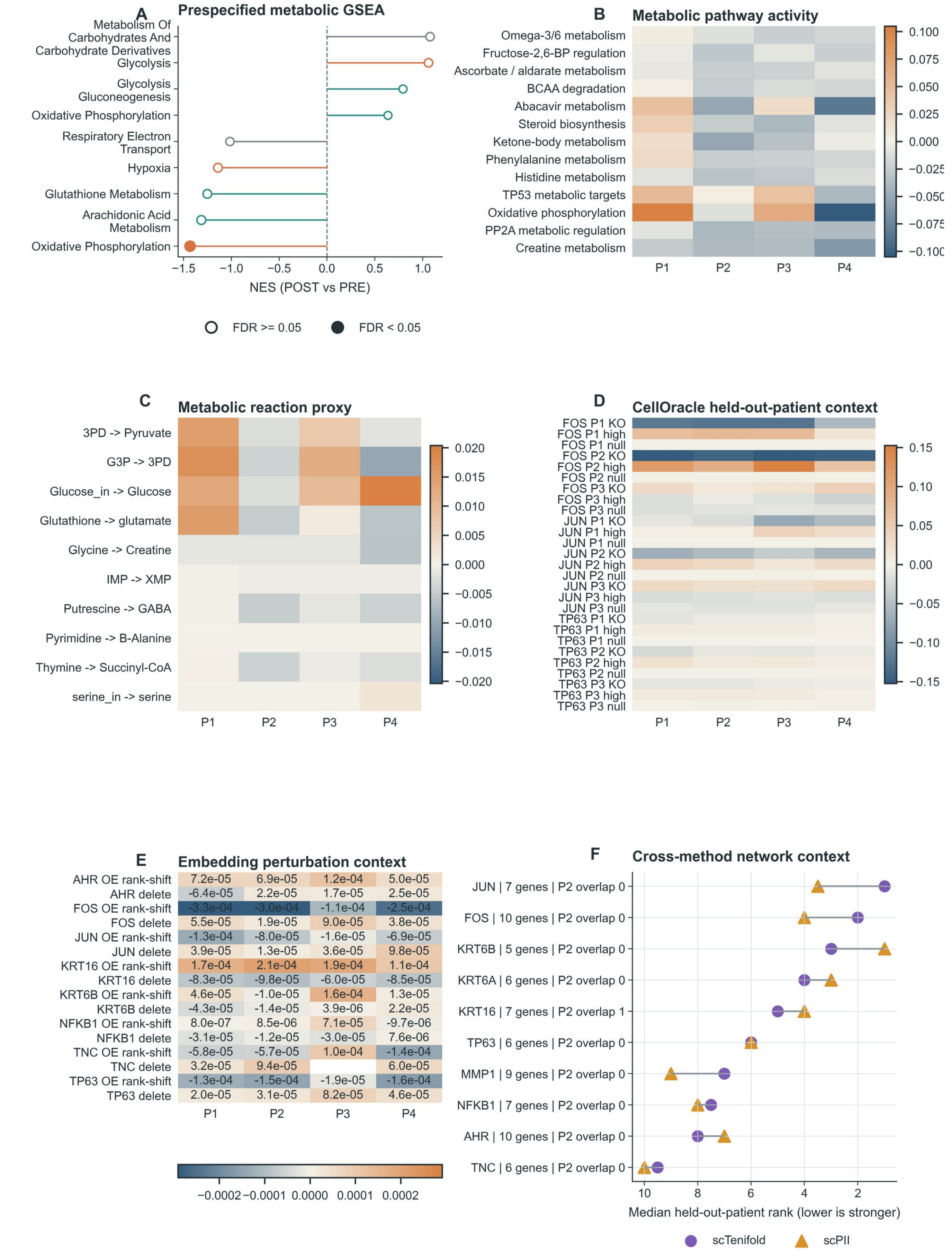

Supplementary Figure S3

Supplementary Figure S3 | Communication and hypertrophic-scar treatment-context analyses. a, Global CellChat interaction architecture, ligand–receptor communication probabilities, paired source–target changes, pathway information flow, incoming and outgoing signaling roles, and communication candidates that did not meet the prespecified criteria. b, Stable HTS fibroblast states and marker expression, the complete six-program patient/donor effect matrix and source-level pathway comparison. c, GSE320017 quality-control retention, resolution stability, state-annotation stability, state representation, fixed-program localization and reciprocal signature context. Global CellChat results are interpreted as architecture-level context; patient-level communication inference remains restricted to the nine axes meeting all prespecified evidence criteria.

Supplementary Figure S3a | Extended intercellular communication context

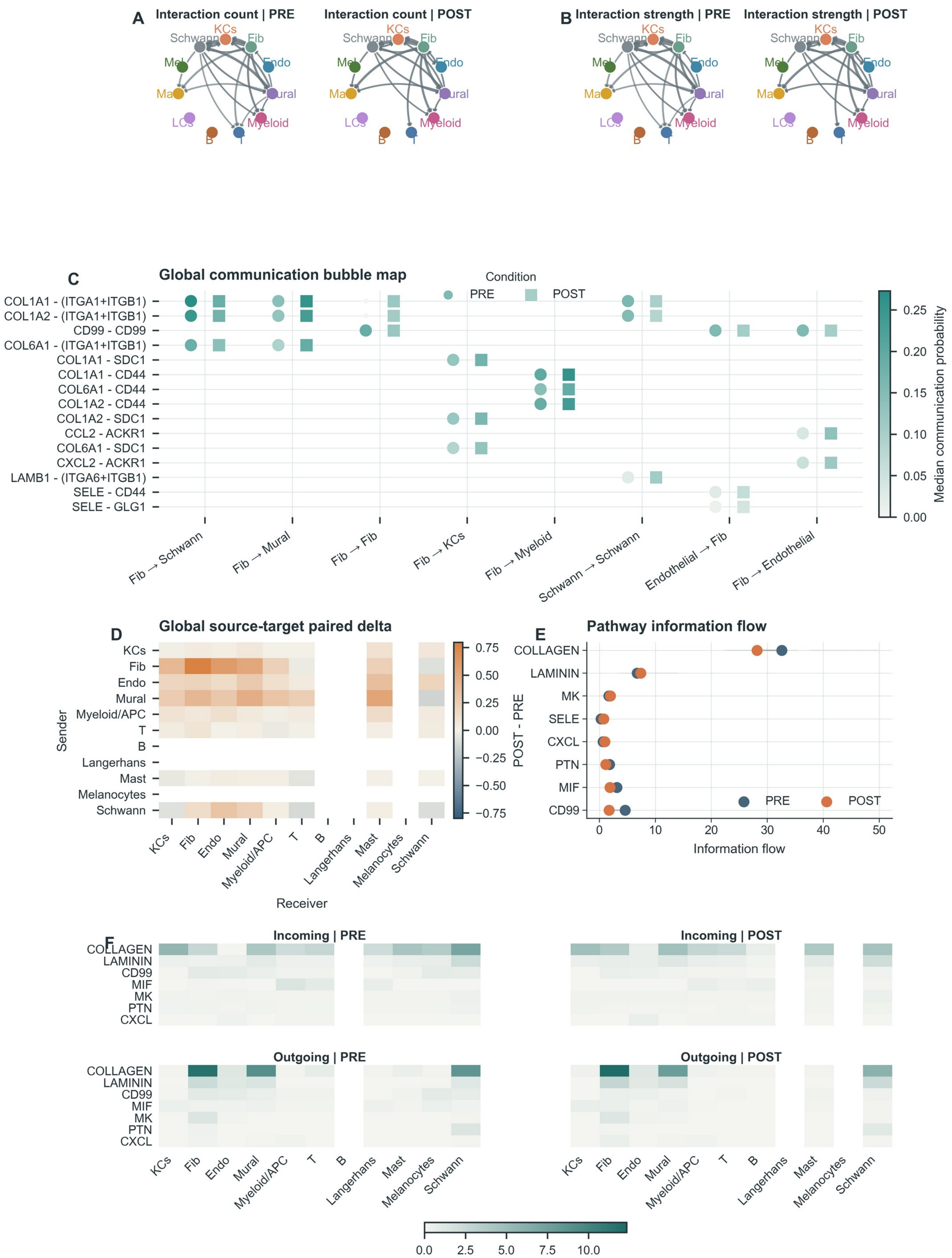

Median role centrality

G

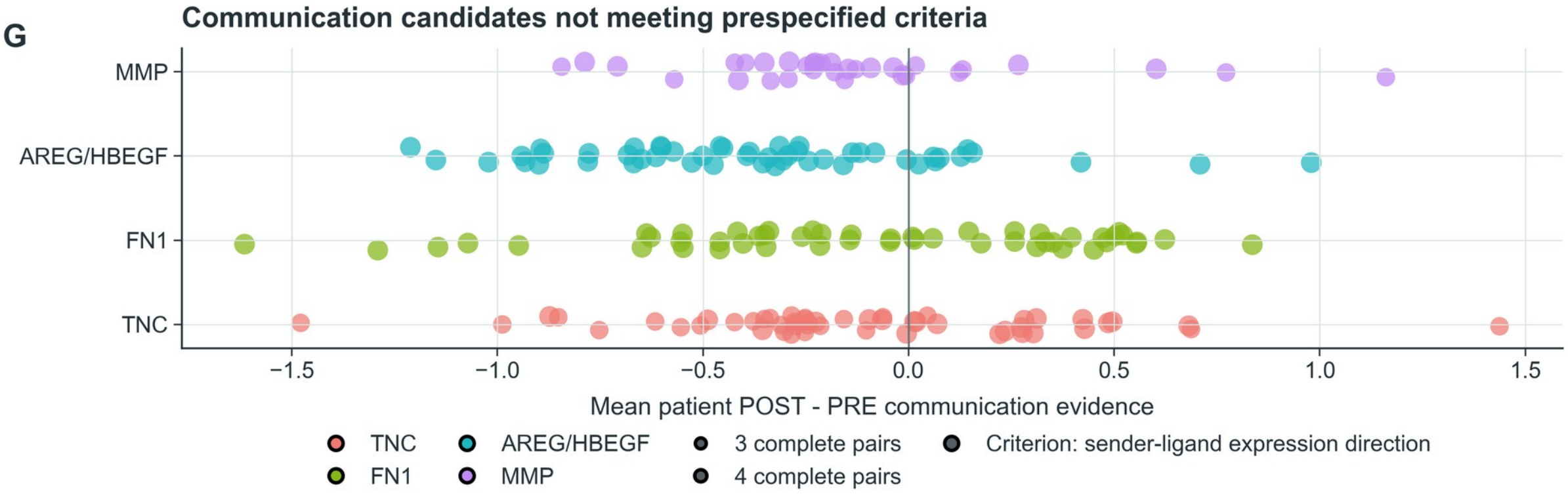

Supplementary Figure S3b | Extended fixed-program and pathway context

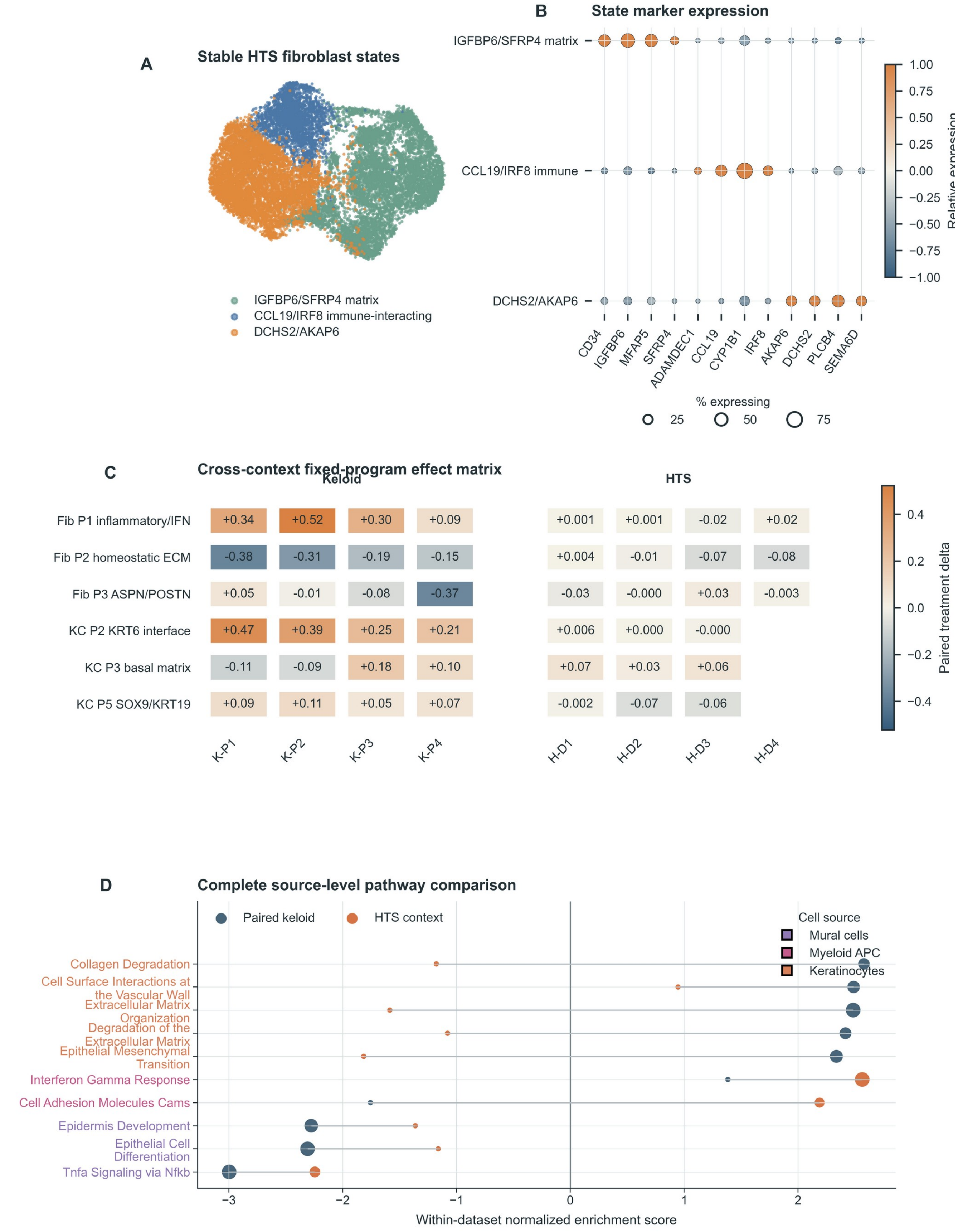

Supplementary Figure S3c | GSE320017 quality control and stability

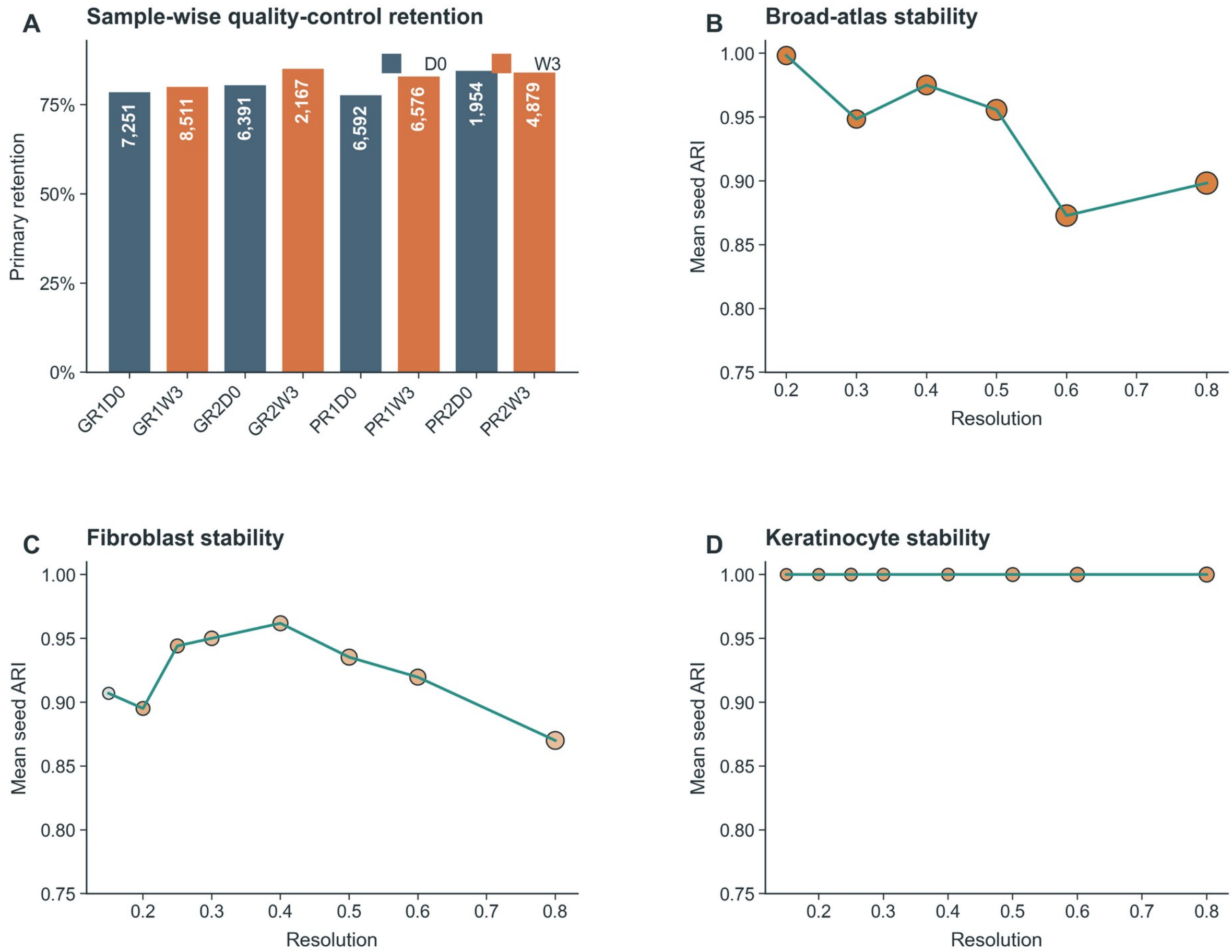

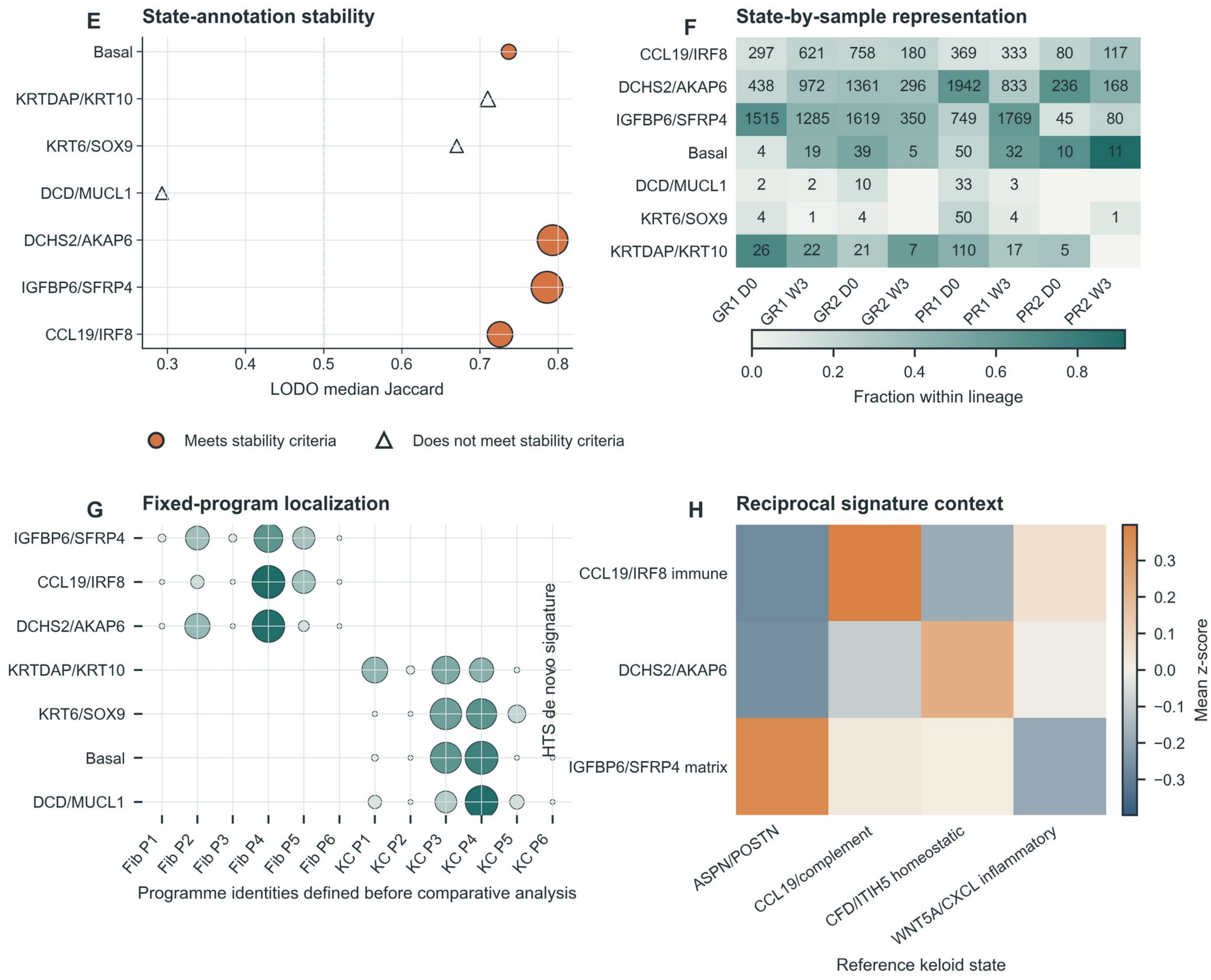
E
State-annotation stability
Basal
KRTDAP/KRT10
KRT6/SOX9
DCD/MUCL1
DCHS2/AKAP6
IGFBP6/SFRP4
CCL19/IRF8
0.3
0.4
0.5
0.6
0.7
0.8
LODO median Jaccard
Meets stability criteria
Does not meet stability criteria
F
State-by-sample representation
CCL19/IRF8 297 621 758 180 369 333 80 117
DCHS2/AKAP6 438 972 1361 296 1942 833 236 168
IGFBP6/SFRP4 1515 1285 1619 350 749 1769 45 80
Basal 4 19 39 5 50 32 10 11
DCD/MUCL1 2 2 10 33 3
KRT6/SOX9 4 1 4 50 4 1
KRTDAP/KRT10 26 22 21 7 110 17 5
GR1 D0
GR1 W3
GR2 D0
GR2 W3
PR1 D0
PR1 W3
PR2 D0
PR2 W3
0.0
0.2
0.4
0.6
0.8
Fraction within lineage
G
Fixed-program localization
IGFBP6/SFRP4
CCL19/IRF8
DCHS2/AKAP6
KRTDAP/KRT10
KRT6/SOX9
Basal
DCD/MUCL1
HTS de novo signature
Fib P1
Fib P2
Fib P3
Fib P4
Fib P5
Fib P6
KC P1
KC P2
KC P3
KC P4
KC P5
KC P6
Programme identities defined before comparative analysis
H
Reciprocal signature context
CCL19/IRF8 immune
DCHS2/AKAP6
IGFBP6/SFRP4 matrix
ASPN/POSTN
CCL19/complement
CFD/ITIH5 homeostatic
WNT5A/CXCL inflammatory
Reference keloid state
0.3
0.2
0.1
0.0
−0.1
−0.2
−0.3
Mean z-score

Supplementary Figure S4

Supplementary Figure S4 | Figure 7 tissue quantification. Patient-level PRE and POST values are shown for CK6-family overlap within CK14-positive epithelium, MMP10 overlap within CK14-positive epithelium, CK6-family/CK14/MMP10 triple overlap and CD74-positive cell density. Regions of interest were technical replicates, and patient was the inferential unit. The older CK14/COL4/LYZ assay was used only for qualitative spatial context and was not quantified.

Supplementary Figure S4 | Figure 7 tissue quantification

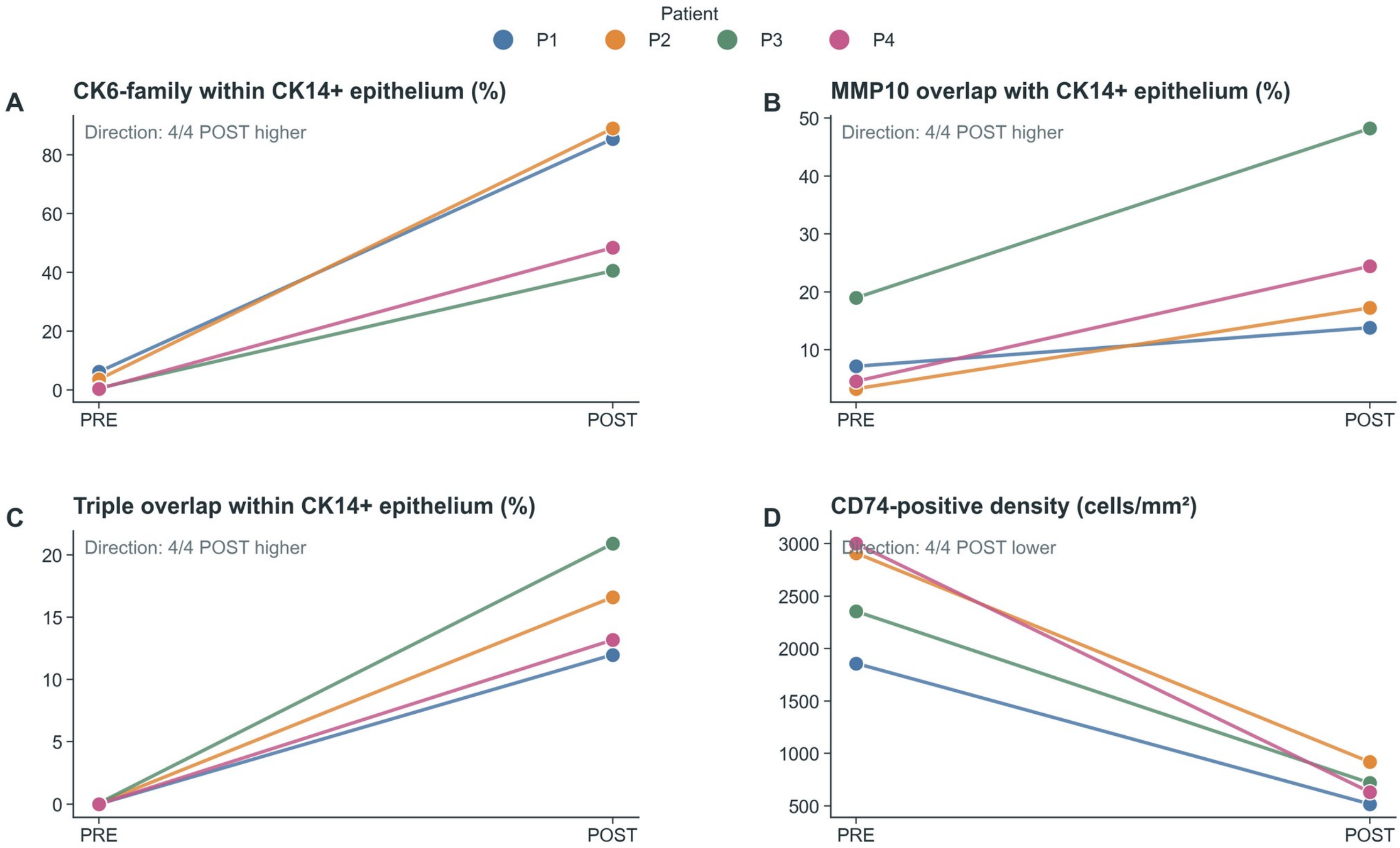

ROI values were aggregated within each patient-condition pair; ROI were technical replicates and patient was the inferential unit.

Supplementary tables

Supplementary Table S1

Supplementary Table S1 | Analysis units, sample-specific quality-control thresholds and broad cell-class counts.

The complete table is provided in worksheet Table S1 of 05_Supplementary_Tables.xlsx.

Supplementary Table S2

Supplementary Table S2 | Patient-level state and programme changes, selected differentially expressed genes and pathway summaries for Figures 1–4.

The complete table is provided in worksheet Table S2 of 05_Supplementary_Tables.xlsx.

Supplementary Table S3

Supplementary Table S3 | Communication axes meeting all prespecified evidence criteria, candidates not meeting those criteria and within-lineage GSE320017 treatment-context summaries.

The complete table is provided in worksheet Table S3 of 05_Supplementary_Tables.xlsx.

Supplementary Table S4

Supplementary Table S4 | Figure 7 deidentified region-level and patient-level values, endpoint definitions and paired statistical summaries.

The complete table is provided in worksheet Table S4 of 05_Supplementary_Tables.xlsx.
